# Dissemination and persistence of antimicrobial resistance (AMR) along the wastewater-river continuum

**DOI:** 10.1016/j.watres.2024.122204

**Published:** 2024-08-03

**Authors:** Daniel S. Read, H. Soon Gweon, Michael J. Bowes, Muna F. Anjum, Derrick W. Crook, Kevin K. Chau, Liam P. Shaw, Alasdair Hubbard, Manal AbuOun, Holly J. Tipper, Sarah J. Hoosdally, Mark J. Bailey, A. Sarah Walker, Nicole Stoesser, on behalf of the REHAB consortium, Manal AbuOun, Muna F. Anjum, Mark J. Bailey, Leanne Barker, Brett H, Mike J. Bowes, Kevin K. Chau, Derrick W. Crook, Nicola de Maio, Nicholas Duggett, Daniel J. Wilson, Sophie George, Daniel Gilson, H. Soon Gweon, Alasdair Hubbard, Sarah J. Hoosdally, William Matlock, James Kavanagh, Hannah Jones, Timothy E. A. Peto, Hayleah Pickford, Daniel S. Read, Robert Sebra, Liam P. Shaw, Anna E. Sheppard, Richard P. Smith, Emma Stubberfield, Nicole Stoesser, Jeremy Swann, A. Sarah Walker, Neil Woodford

**Affiliations:** a UK Centre for Ecology & Hydrology (UKCEH), Benson Lane, Crowmarsh Gifford, Wallingford OX10 8BB, UK; b School of Biological Sciences, University of Reading, Reading, UK; c Department of Bacteriology, Animal and Plant Health Agency, Addlestone, Surrey KT15 3NB, UK; d Nuffield Department of Medicine, University of Oxford, Oxford, UK; e Department of Biology, University of Oxford, 11a Mansfield Road, Oxford OX1 3SZ, UK; f Department of Biosciences, Nottingham Trent University, Nottingham NG11 8NS, UK

**Keywords:** Antimicrobial resistance, Resistome, Wastewater, River, Sediment

## Abstract

Antimicrobial resistance (AMR) is a global health hazard. Although clinical and agricultural environments are well-established contributors to the evolution and dissemination of AMR, research on wastewater treatment works (WwTWs) has highlighted their potential role as disseminators of AMR in freshwater environments. Using metagenomic sequencing and analysis, we investigated the changes in resistomes and associated mobile genetic elements within untreated wastewater influents and treated effluents of five WwTWs, and sediments collected from corresponding river environments in Oxfordshire, UK, across three seasonal periods within a year. Our analysis demonstrated a high diversity and abundance of antimicrobial resistance genes (ARGs) in untreated wastewater influents, reflecting the varied anthropogenic and environmental origins of wastewater. WwTWs effectively reduced AMR in the final effluent, with an average 87 % reduction in normalised ARG abundance and an average 63 % reduction in richness. However, wastewater effluents significantly impacted the antimicrobial resistome of the receiving rivers, with an average 543 % increase in ARG abundance and a 164 % increase in richness from upstream sediments to downstream sediments. The normalised abundance of the human gut-associated bacteriophage crAssphage was highly associated with both ARG abundance and richness. We observed seasonal variation in the resistome of raw influent which was not found in the effluent-receiving sediments. We illustrate the potential of WwTWs as focal points for disseminating ARGs and resistance-selecting chemicals, contributing to the elevation of environmental AMR. Our study emphasises the need for a comprehensive understanding of the anthropogenic impacts on AMR evolution and dissemination in wastewater and river environments, informing efforts to mitigate this growing public health crisis.

## 1 Introduction

Antimicrobial resistance (AMR) poses a substantial threat to human health and well-being (Murray et al., 2022). Although antibiotic usage in clinical and food production settings is recognised as a primary driver of AMR, anthropogenic interactions with the environment play a crucial role in the evolution and spread of AMR (Larsson and Flach, 2022; Pradier and Bedhomme, 2023). Human activity can contaminate the environment with antimicrobial-resistant bacteria (ARB), antimicrobial resistance genes (ARGs), and a wide range of anthropogenic chemicals that have the potential to select for resistance in microbial communities or increase the rates of horizontal gene transfer (HGT) (Wang et al., 2019). These activities can create opportunities for the onward environmental transmission of ARB (Stanton et al., 2020) or the generation of new variants or mechanisms of resistance in clinically relevant pathogens via HGT (Larsson et al., 2018).

Wastewater is considered a potential hotspot for AMR evolution and dissemination because of its diverse microbial and chemical environments, both within wastewater distribution networks and wastewater treatment works (WwTWs) (Smalla et al., 2018). The wastewater microbiome is composed of microbes from human, animal, and environmental sources and contains a high diversity of antimicrobials and other pharmaceutical compounds, potentially creating an environment conducive to the evolution and selection of AMR (Manaia et al., 2018). Because treated wastewater effluent is typically released into the environment, WwTWs may act as focal points for the dissemination of ARB and resistance-selecting chemicals, elevating AMR in the environment, and increasing the risk of HGT and the transmission of AMR to humans, livestock, and wildlife populations (Stanton et al., 2020).

Untreated wastewater influents have been shown to exhibit high diversity and abundance of ARGs as well as a high frequency and abundance of genetic elements involved in the mobilisation of genes, such as insertion sequences (ISs), relative to other environments (Berglund et al., 2023). WwTWs have previously been shown to be effective at reducing the absolute concentrations of both ARB and ARGs in effluents (Ju et al., 2019; Quintela-Baluja et al., 2019), although consistent patterns of ARB and ARG removal are difficult to identify in the literature because of the wide range of WwTW processes and sample types that have been studied, as well as inconsistent methodologies such as culturing and targeted and non-targeted (meta)genomics (Pazda et al., 2019).

Rivers and streams are frequent recipients of both treated and untreated wastewater and host diverse natural microbial communities (Read et al., 2015) that originate from terrestrial, aquatic, subsurface, and host-associated sources (Newton et al., 2013). Pristine environmental microbial communities, which include those found in water, sediments, and soils, contain a natural "background" level of AMR (i.e., AMR that is not associated with selective pressure from human use of AMR-driving chemicals), although frequently observed at lower levels than in anthropogenically impacted environments (Pruden et al., 2012) and are generally not dominated by clinically relevant ARG variants (Zhou et al., 2018). Although wastewater effluents are one of the primary drivers of elevated AMR in water and sediments within river networks (Pruden et al., 2012), there are numerous other potential sources of ARB, ARGs, and selective anthropogenic chemicals, including livestock farming, urban runoff, and one-off anthropogenic pollution events (Amos et al., 2015; Karkman et al., 2019).

The full extent of the role played by rivers and streams as receptors and dispersers of treated wastewater effluent and the ARB, ARGs, and AMR-selective chemicals they contain is not yet comprehensively understood (Larsson et al., 2018). Critical knowledge gaps exist concerning the dynamics of the persistence and longitudinal (downstream) and vertical (into sediments) transport of ARB and ARGs within rivers and streams following wastewater discharge. Rivers and wastewater are highly dynamic environments, and seasonally driven changes in flow and organic matter input can cause fluctuations in biology, pH, dissolved organic matter, and nutrients (Bowes et al., 2016). There is a need to understand how these factors interact with the persistence and dissemination of AMR to comprehend the impact of wastewater on these receiving environments.

This study aimed to understand whether there were changes in the antimicrobial resistome distribution along a continuum of untreated influent to treated wastewater effluent to river sediments. To identify universal responses across different WwTWs and rivers and over seasons, five replicate WwTWs and corresponding rivers in Oxfordshire, UK, were sampled over a year during three seasonal sampling events. We used metagenomic analysis to examine wastewater influent, effluent, and sediments from the receiving rivers. We assessed the impact of WwTW treatment (from influent to effluent) on the composition, diversity, and abundance of the resistome, along with related factors, such as ISs and plasmids (genetic elements that can be involved in the transmission of AMR). We also evaluated the effects of WwTW effluent on the longitudinal dissemination of AMR in the receiving rivers and the impact of seasonality on the antimicrobial resistome in these niches and dissemination across this continuum.

## 2 Material and methods

### 2.1 Sampling and physicochemical analysis

**Fig. 1:**
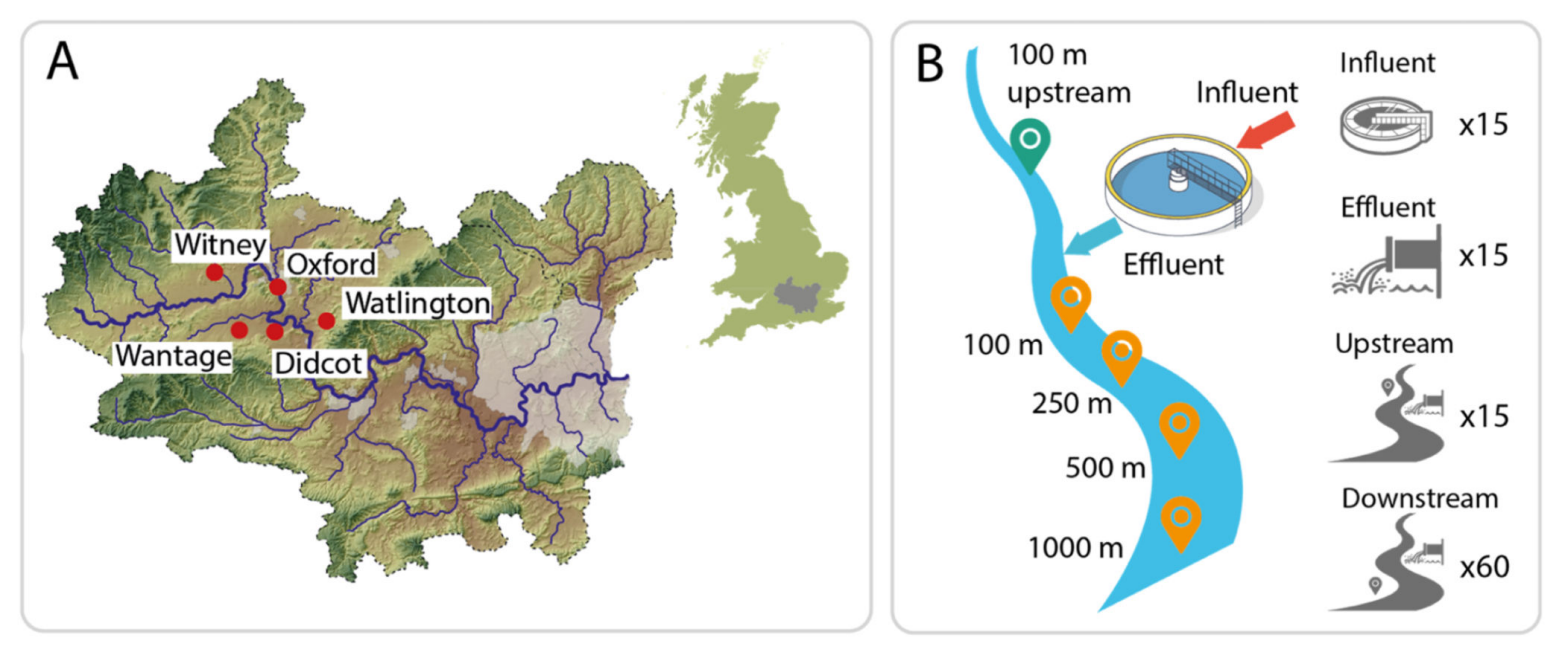
(A) Locations of the five wastewater treatment works, Oxford, Didcot, Witney, Wantage, and Watlington, within the River Thames, UK catchment. (B) Schematic of the sediment sampling sites at each location, showing representative locations of samples taken from the wastewater influent, wastewater effluent, and river sites upstream and downstream of the wastewater effluent point source.

To evaluate the structure and variation of AMR among WwTWs and their corresponding receiving rivers, a total of five sites were chosen based on their geographical location within the Oxfordshire Clinical Commissioning Group (CCG) boundary, WwTW treatment processes, wastewater Population Equivalent (PE) served, WwTW consented flow, lack of upstream WwTW inputs, and accessibility of the effluent receiving river for sampling upstream and downstream of the effluent point source. The details of the WwTWs are shown in Table 1, and the corresponding locations of the sites are illustrated on a map in Fig. 1A. Maps of individual sites are shown in Supplementary Fig. S1A–S1E.

All five WwTWs and rivers were sampled in 2017 over three sampling rounds, from February to March, June to July, and October to November. Sewage influent samples were collected after the WwTW coarse screens, and effluent samples were collected at the last effluent sampling point within the treatment works, before entering the river. For each sampling round, repeated (between 4 and 6) 200 mL grab samples of influent and effluent were collected in a six-hour period between 9am and 12pm using an extendable sampling pole and sterile Whirl-Pak™ collection bags. Repeat samples from each sampling round were pooled during processing to reduce the known impact of temporal variability on the wastewater flow and microbial/AMR composition (Chau et al., 2022). The river sampling points were ca. 100 m and 10 m upstream, and ca. 10 m, 100 m, 250 m, 500 m and 1000 m downstream of the effluent entry point into each river (Fig. 1B). Water samples for nutrient chemistry were collected from all these points using 2 L HDPE plastic bottles at the end of a sampling pole from the centre of the river channel. The water nutrient chemistry procedures are described in the Supplementary methods.

Sediment samples (*n* = 75) were collected from a subset of river sampling locations, including 100 m upstream, and 100 m, 250 m, 500 m and 1000 m downstream of the WwTWs, using a custom aluminium sampling pole that held a removable, 50 mL polypropylene centrifuge tube. Using a new, sterile 50 mL tube for each sample, sediment was collected from the top ~5 cm of the surface sediment layer at three points at each sampling location (left bank, centre of the river, and right bank, looking downstream) to account for in-river spatial variability in sediment composition. Sediment samples were stored in the dark in an insulated box at 4 °C until they were returned to the laboratory (<6 h), where they were stored at −20 °C until analysis.

### 2.2 Sample processing and DNA extraction

Sewage and sediment samples were processed as described in the supplementary methods, and DNA extraction was performed using the Qiagen PowerSoil kit (Qiagen, UK), following the manufacturer’s instructions

500 ng of DNA from each sample was used for library preparation. Libraries were constructed using the NEBNext Ultra DNA Sample Prep Master Mix Kit (NEB) with minor modifications and a custom automated protocol on a Biomek FX (Beckman) (described in Lamble et al. 2013). DNA sequencing was performed on an Illumina HiSeq4000, generating approximately 80 M, 150 bp paired-end reads per sample (24Gbp).

### 2.3 Screening for microorganic contaminants

In the October–November sampling round, effluent samples were collected for screening of micro-organics using non-target liquid chromatography/mass spectrometry (LCMS) analysis. One litre of water sample was collected 100 m upstream, 100 m downstream and effluent using 1 L brown glass bottles with a PTFE liner. The samples were shipped on ice in an insulated shipping box to the National Laboratory Service Exeter for the analysis of 686 polar organic compounds using the time-of-flight (Q-TOF) LC/MS method, as described by White et al. (2016).

### 2.4 DNA sequence processing: metagenomics

DNA sequence data were processed using the AMR analysis pipeline ‘ResPipe’ (Gweon et al., 2019). Briefly, taxonomic classification was performed using Kraken2 (Wood et al., 2019). AMR gene counts were generated for sequences that were mapped with 100 % sequence identity against the Comprehensive Antibiotic Resistance Database v.3.0.9 (Jia et al., 2017). Enterobacterales plasmids and Insertion Sequence counts were mapped with 100 % sequence identity against a curated dataset of complete Enterobacteriaceae plasmids compiled from the NCBI nucleotide database (Orlek et al., 2017) and the ISfinder database (Siguier et al., 2006). We focused on Enterobacteriaceae plasmids as this family is an important component of intestinal microbiota and a significant causative agent in hospital-acquired and community-acquired infections. The proliferation and dissemination of multidrug-resistant (MDR) Enterobacteriaceae strains have significantly constrained available therapeutic interventions. The resulting tables were normalised to Fragments Per Kilobase Million (FPKM), gene length and 31 single-copy genes to give an estimation of ‘genes per cell’ (Yin et al., 2023), as described in the Supplementary Methods.

### 2.5 Statistical analyses and visualisation

All statistical analyses and visualisations were performed using R programming language version 4.4.2. (R Core Team, 2018) in RStudio (version 2022.12.0 + 353). Non-metric multidimensional scaling (NMDS) was conducted using the R package ‘Vegan’ (version 2.6–4) (Dixon, 2003) using Bray-Curtis distances. PERMANOVA, using the function ‘Adonis’ in Vegan, was used to test for significant differences between groups (e.g., between sediment sampling locations and seasonal or site-based differences) using 999 permutations. ARG richness and abundance data were tested for normality using the shapiro_test function from ‘rstatix’ (version 0.7.2) (Kassambara, 2023a) and the ggqqplot function in ‘ggpubr’ (version 0.6.0) (Kassambara, 2023b). When the data were not normally distributed, log transformation was used to approach normality. An ANOVA or Kruskal-Wallis test was used to test for global differences between groups, and a T-test or Wilcoxon test was used to test for differences between upstream sites and paired downstream sites, using the compare_means function in ‘ggpubr’. Linear regressions were used to examine the relationships between AMR gene richness and abundance, and the distance downstream from the effluent point source. DESeq2 (Love et al., 2014) was used to test for differentially abundant genes between pairs of sample types (e.g. influent versus effluent and upstream 100 m versus downstream 100 m) using a significance level of *P* = 0.001. To test for a relationship between AMR composition and distance downstream, the ARG distance matrix was plotted against an Euclidian distance matrix of distance downstream and tested for statistical significance using a Mantel test in Vegan. The fast expectation-maximisation for microbial source tracking (FEAST) algorithm in R (Shenhav et al., 2019) was used to estimate the contribution of influent wastewater resistomes to effluent and sediment resistomes on a site-by-site basis. The R package EulR (Larsson, 2022) was used to plot an Euler diagram showing the overlap between ARGs from wastewater influent, effluent, and upstream and downstream sediments, and https://www.rawgraphs.io was used to create a circle plot to summarise unique ARGs associated with each environmental compartment. The package ‘UpSetR’ (Conway et al., 2017) was used to create UpSet overlap plots to examine the overlap between ARGs, ISs and Enterobacterales plasmids across seasons.

In the context of AMR, PNECs are thresholds thought to indicate the potential to select for resistance in microorganisms (AMR Industry Alliance, 2023). Risk quotients (RQs) can be calculated from the ratio of measured environmental concentrations (MECs)/PNEC and indicate the scale of PNEC exceedance (Sengar and Vijayanandan, 2022). To evaluate the potential risk of selection of AMR associated with the concentrations of antibiotics measured in this study, RQs were calculated using the lowest PNEC collated in the AMR Industry Alliance list of PNECs for antibiotic discharge targets. These included PNEC-Environment (PNEC-Env) values (Brandt et al., 2015; Le Page et al., 2017) and PNEC-Minimum inhibitory concentration (PNEC-MIC) values (AMR Industry Alliance, 2023; Bengtsson-Palme and Larsson, 2016). RQs were only calculated for antibiotics with available PNEC values from the AMR Industry Alliance (2023). For AMR, RQs > 1 indicate a significant risk of AMR development (Sengar and Vijayanandan, 2022).

## 3 Results

### 3.1 Patterns of AMR in wastewater and rivers

**Fig. 2:**
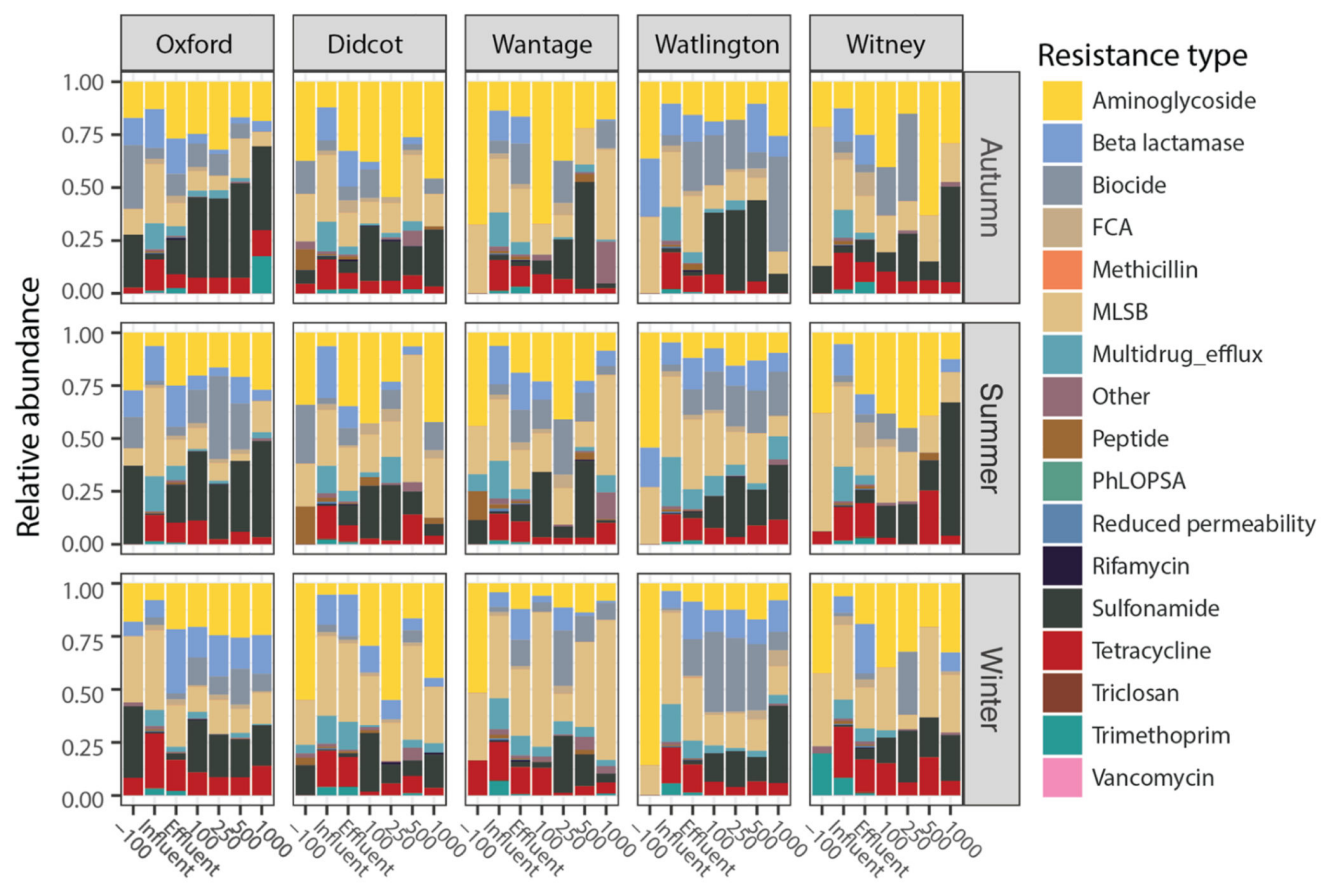
The relative abundance of AMR gene families was grouped according to the resistance categories. Abbreviations: FCA = fluoroquinolone, quinolone, florfenicol, chloramphenicol, and amphenicol. MLSB = macrolide-lincosamide-streptogramin B. PhLOPSA = Phenicols, Lincosamides, Oxazolidinones, Pleuromutilins, and Streptogramin A, Other = grouped low abundance categories.

To understand the role of WwTWs and effluent-receiving rivers in ARG distribution and dissemination, we performed deep sequencing metagenomics on 105 samples representing sewage influent (*n* = 15), effluent (*n* = 15), and upstream and downstream sediment (*n* = 75) at five WwTW/river locations over three seasonal sampling points. Fig. 2 shows an overview of the antimicrobial resistome composition from all samples across WwTWs, river sediment and seasonal sampling frames.

**Fig. 3:**
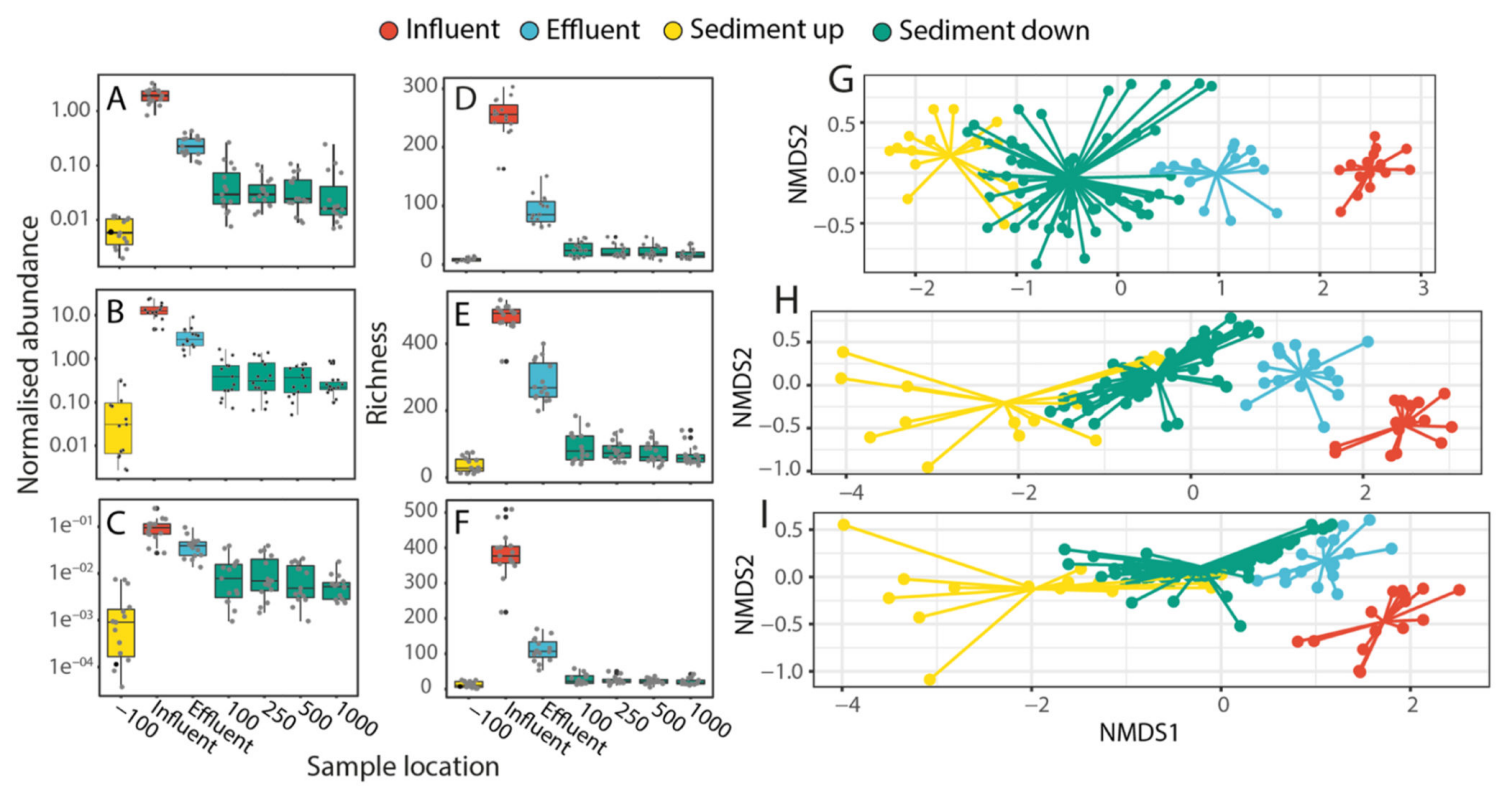
Box plots showing the normalised abundance (genes per cell) of; (A) ARGs, (B) insertion sequences, (C) Enterobacterales plasmids, and the richness of; (D) ARGs, (E) insertion sequences, and (F) Enterobacterales plasmids, from upstream sediment, influent, effluent, and downstream sediment samples aggregated across sites. Non-metric multidimensional scaling plots showing the relationship between samples based on (G) composition of ARGs, (H) insertion sequences (ISs), and (I) Enterobacterales plasmids.

The normalised abundance of ARGs varied considerably across the different sampled compartments (Fig. 3A), with the highest average abundance found in raw wastewater influent (mean = 1.954, SD = 0.630, *n* = 15), followed by the final effluent (mean = 0.247, SD = 0.102, *n* = 15), downstream sediments (mean = 0.045, SD = 0.050, *n* = 60), and finally upstream sediments (mean = 0.007, SD = 0.004, *n* = 15). This represents an average reduction of 87 % in normalised ARG abundance caused by wastewater treatment, a further 82 % reduction in abundance from sewage effluent to sediments in receiving rivers, and a 543 % increase in abundance from upstream sediments to downstream sediments that were exposed to sewage effluents.

Similar patterns were observed in the richness of ARGs (Fig. 3D), with the highest ARG richness found in the raw wastewater (mean = 252.73, SD = 33.07, *n* = 15), followed by the final effluent (mean = 92.60, SD = 24.88, *n* = 15), downstream sediments (mean = 21.98, SD = 10.75, *n* = 60), and upstream sediments (mean = 8.33, SD = 3.35, *n* = 15). This represents an average (across all sites) reduction of 63 % in ARG richness caused by wastewater treatment, a further 76 % reduction in richness from sewage effluent to sediments in receiving rivers, and a 164 % increase in richness from upstream sediments to downstream sediments that were exposed to sewage effluents. In total, 298 unique ARGs conferring resistance to 15 antibiotic classes were identified (Supplementary data S2 and S3). The most common resistance genes belonged to the beta-lactamase (*n* = 136 ARGs), multidrug efflux (*n* = 95), aminoglycoside (*n* = 70), fluoroquinolone, quinolone, florfenicol, chloramphenicol, and amphenicol (FCA) (*n* = 54), macrolide-lincosamide-streptogramin B (MLSB) (*n* = 49), peptide (*n* = 40), tetracycline (*n* = 37), trimethoprim (*n* = 17) resistance classes.

There was a significant difference in both AMR richness and normalised abundance of ARGs between the 100 m upstream site and the 100 m, 250 m, 500 m, and 1000 m downstream sites (Supplementary Fig. S2A, S2B; Richness *t*-test; p < 0.001. Abundance Wilcoxon test; p < 0.05). When all locations were analysed together, there was a significant (although weak) negative correlation between log AMR gene richness and downstream distance (Supplementary Fig. S3, linear regression; adj-R^2^ = 0.06, *p* < 0.05), indicating a declining impact further away from the effluent source. This trend was not observed for ARG abundance.

Differences in the richness and normalised abundance of insertion sequences and Enterobacterales plasmids were also observed across sample types (wastewater influent, effluent, and river sediments (ISs: Fig. 3B and 3E; Enterobacterales plasmids: Fig. 3C and 3F). Wastewater influent had a significantly higher richness and abundance of ISs and Enterobacterales plasmids than the effluent and sediment samples (Richness; Wilcoxon test p ≤ 0.001, Abundance; Wilcoxon test p ≤ 0.001). Effluent had a significantly higher richness and abundance of ISs and Enterobacterales plasmids than the sediment samples (Richness; Wilcoxon test p ≤ 0.001, Abundance; Wilcoxon test p ≤ 0.001). Notably, sediments sampled up to 1 km downstream from each WwTW had elevated richness and abundance of ARGs, ISs, and Enterobacterales plasmids compared with sediments sampled upstream of WwTWs.

NMDS and Adonis analyses of the ARGs, insertion sequences, and Enterobacterales plasmids (Fig. 3G–3I) showed significant differences in composition between influent, effluent, upstream sediment, and downstream across all WwTW locations and receiving rivers (Adonis; ARG F = 15.27, R^2^ = 0.23, p ≤ 0.001, insertion sequences F = 28.13, R^2^ = 0.36, p ≤ 0.001, plasmids F = 28.79, R^2^ = 0.36, p ≤ 0.001). This pattern was also observed for ARGs when each of the five WwTW and receiving river locations were analysed individually (Supplementary Fig. S4, Adonis; R^2^ > 0.5, p ≤ 0.001).

### 3.2 AMR across compartments

Samples representing transitions along the wastewater and wastewater-environment continuum were used to identify differentially abundant ARGs, ISs, and Enterobacterales plasmids. These were untreated wastewater versus final effluent and upstream sediments (low/no impact from effluent) versus downstream sediments (high impact from effluent).

**Fig. 4:**
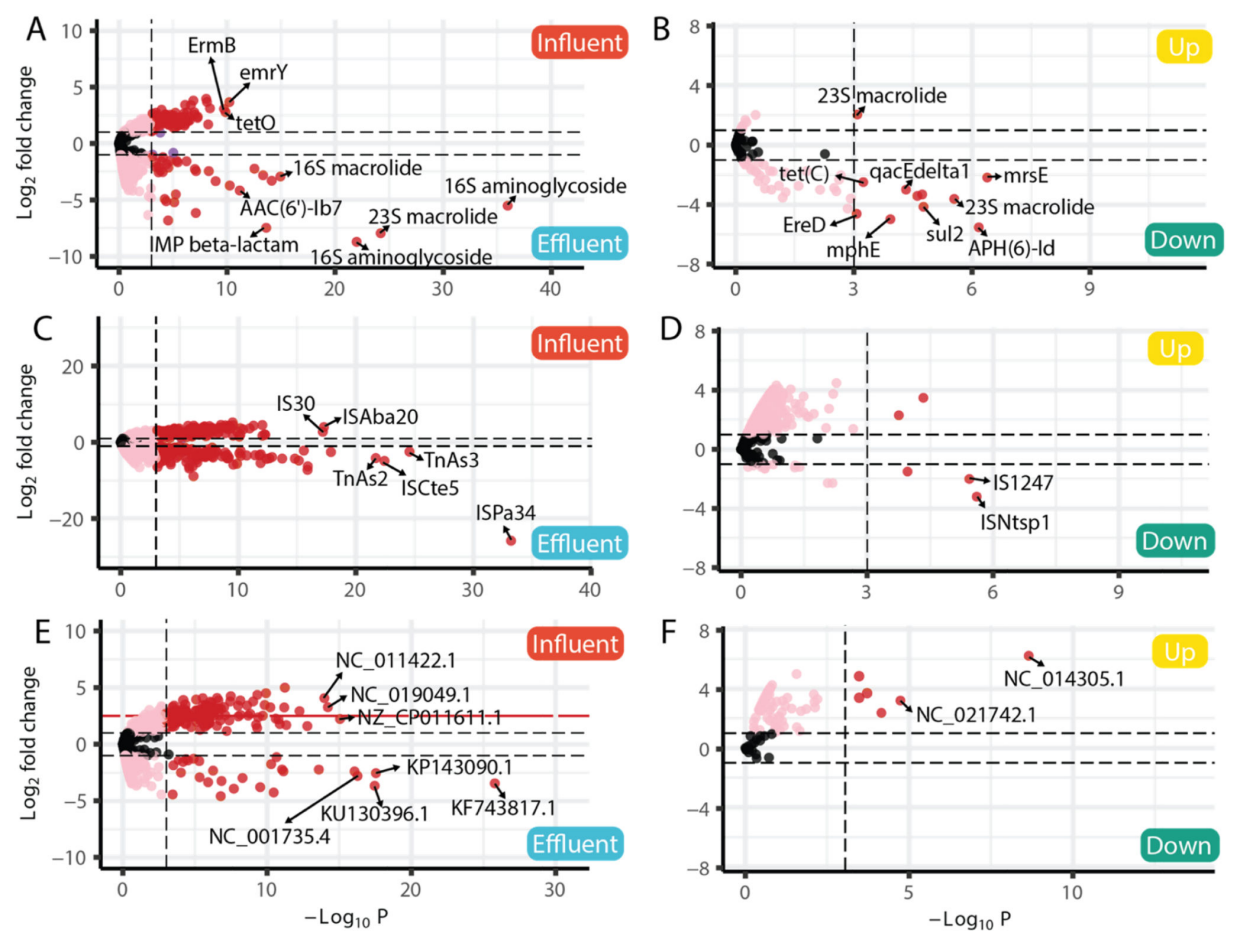
Volcano plots showing differentially abundant: Antimicrobial resistance genes (ARGs) in; (A) untreated influent and treated effluent, and in (B) upstream and downstream sediments. Insertion Sequences (ISs) in; (C) untreated influent and treated effluent, and in (D) upstream and downstream sediments. Enterobacterales plasmids in; (E) untreated influent and treated effluent, and in (F) upstream and downstream sediments. The vertical dotted line represents a P-value of 0.001.

ARGs found to be elevated in raw influent compared to final effluent included *emrY* (multidrug transport linked to tetracycline resistance, penam, and fluoroquinolone), *ermB* (erythromycin resistance) and *tetO* (tetracycline resistance) (Fig. 4A). ARGs enriched in final effluents included 23S and 16S rRNA mutations conferring resistance to macrolide antibiotics, 16S rRNA mutations conferring resistance to amino-glycoside antibiotics, *bla*_IMP_ (a broad-spectrum metallo-beta-lactamase/carbapenemase), and *AAC(6*′*)-Ib7* (a plasmid-encoded aminoglycoside acetyltransferase) (Fig. 4A).

Examples of ISs that were more abundant in raw influent compared to final effluent included IS*Aba20* (origin species *Acinetobacter bau-mannii*) and IS*30* (origin species *Escherichia coli*), with insertion sequences IS*Pa34* (origin species *Pseudomonas aeruginosa*), IS*Cte5* (origin species *Comamonas testosteroni*), Tn*As3* and Tn*As2* (origin species *Aeromonas salmonicida*) showing the opposite trend (Fig. 4C).

There were also clear patterns in reads mapping to Enterobacterales plasmids between untreated wastewater and treated effluent across the five WwTWs and three seasonal sampling points. For example, *Salmonella enterica* 404ty plasmid pBSSB1 (NC_011422.1), *E. coli* plasmid pCM959 (NC_019049.1) and *Citrobacter freundii* strain CAV1321 plasmid pKPC_CAV1321–244 (NZ_CP011611.1) were more abundant in untreated wastewater, whereas *E. coli* strain E265 plasmid pHS33 (KP143090.1), *Proteus mirabilis* plasmid R772 (KF743817.1), *E. coli* strain S68 plasmid pS68 (KU130396.1) and *Klebsiella aerogenes* plasmid R751 (NC_001735.4) were more abundant in final effluents (Fig. 4E).

Differences in ARGs, ISs and Enterobacterales plasmids between upstream and downstream river sediments were generally less pronounced than for influent and effluent (Fig. 4B, 4D, 4F). Differentially abundant ARGs included 23S rRNA mutation conferring resistance to macrolides, which was more abundant in upstream sediments, and APH(6)-Id (an aminoglycoside phosphotransferase), *sul2* (sulfonamide resistance), *mphE* (a macrolide phosphotransferase), *ereD* (a macrolide esterase), *tet(C)* (encoding a tetracycline efflux pump), *qacEdelta1* (resistance to antiseptics found in many species), and *msrE* (encoding a msr-type ABC-F protein found in many species) enriched in sediments downstream of wastewater effluents (Fig. 4B). With respect to ISs, IS*1247* (origin species *Xanthobacter autotrophicus*) and IS*Ntsp1* (origin species *Nitrosomonas* sp.) were enriched in downstream sediments, and *Erwinia billingiae* Eb661 plasmid pEB170 (NC_014305.1) and *Serratia liquefaciens* ATCC 27,592 plasmid (NC_021742.1) were enriched in upstream sediments (Fig. 4D and F).

### 3.3 AMR in wastewater-receiving rivers

**Fig. 5:**
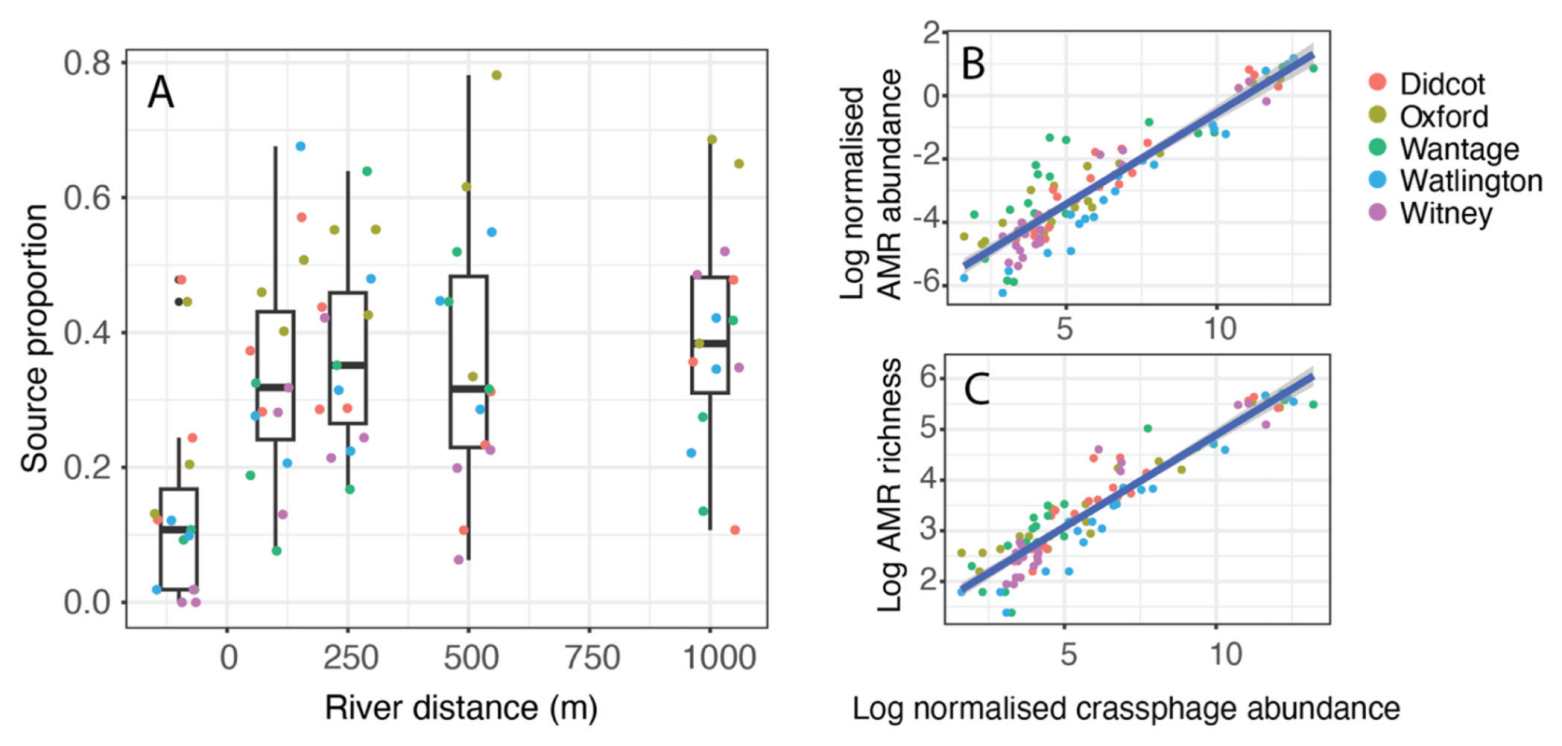
(A) Source estimates of antimicrobial resistance genes (ARGs) from untreated wastewater assigned to each sediment sample from all river sites. For each site, data from three sampling time points are represented. The relationship between the normalised abundance of crAssphage against; (B) normalised ARG abundance and (C) normalised ARG richness, where the lines represent fitted generalised additive models (GAMs) across all sample types (influent, effluent, upstream and downstream sediments).

FEAST analysis showed that the contribution of the untreated wastewater resistome increased downstream of the effluent source (Fig. 5A), moving from an average source proportion of 0.140 (*n* = 15, SD= 0.149) upstream to 0.338 (*n* = 15, SD = 0.164) immediately downstream of the wastewater effluent entry point. There was no decline in the contribution of raw influent to the resistome moving downstream, with a contribution of 0.389 (*n* = 15, SD = 0.165) at the furthest downstream sampling points (1000 m).

The normalised abundance of the human gut-associated bacteriophage crAssphage was found to be highly associated with both the normalised abundance of ARGs (R^2^ = 0.85, F_1, 103_ = 597.4, *p* = <0.001) (Fig. 5B) and AMR richness (R^2^ = 0.88, F_1, 103_ = 795.8, *p* = <0.001) (Fig. 5C) across all sample types (influent, effluent, upstream and downstream sediments). The relationship between crAssphage and ARG abundance and richness was still observed in just the upstream and downstream sediment samples, although less strongly (crAssphage vs. normalised abundance; R^2^ = 0.44, F_1, 73_ = 58.89, *p* = <0.001; crAss-phage vs. ARG richness; R^2^ = 0.61, F_1, 73_ = 119, *p* = <0.001).

**Fig. 6:**
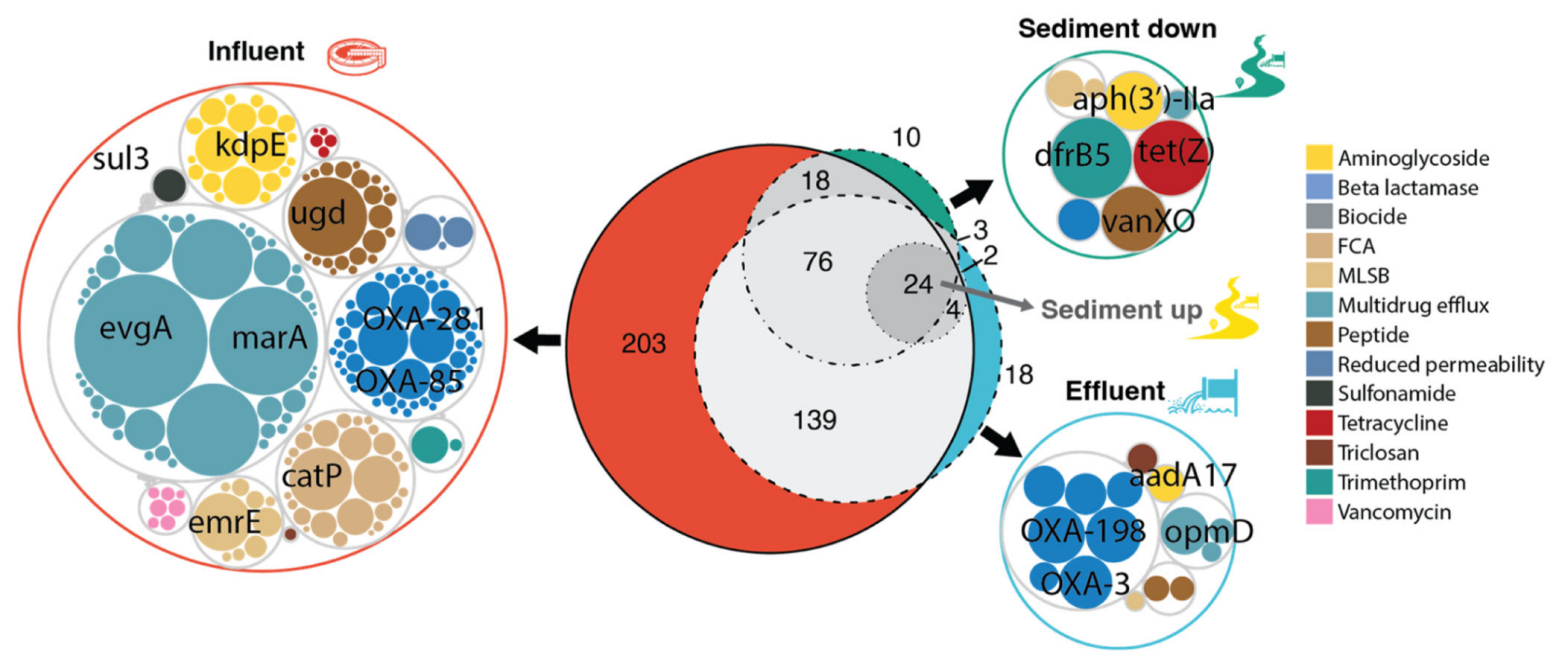
Euler diagram showing the overlap of antimicrobial resistance genes (ARGs) between untreated wastewater (influent), treated wastewater effluent (effluent), and sediments downstream of the effluent entry point to the river, pooled across all sampling locations and time points. Circle packing plots show unique ARGs associated with each environmental compartment. FCA = fluoroquinolone, quinolone, florfenicol, chloramphenicol, and amphenicol. MLSB = macrolide-lincosamide-streptogramin B.

The overlap in ARGs detected in wastewater, treated effluent, and downstream river sediments is shown in Fig. 6, and the ARGs unique to each compartment are listed in Supplementary data S6. The central Euler diagram comprises circles representing the number of ARG types detected in each compartment and the degree of overlap between each compartment. There was a high degree of overlap in ARGs detected in different compartments, with effluent and sediment ARG composition primarily being found within untreated influent. The associated circle plots show ARGs that are unique to the influent, effluent, and downstream sediment samples (no unique ARGs were detected in the upstream sediments). Untreated influent had the highest number of unique ARGs (203), followed by treated effluent (18) and downstream sediments (10), indicating a loss of ARGs during wastewater treatment processes.

### 3.4 Variability in AMR across seasons

**Fig. 7:**
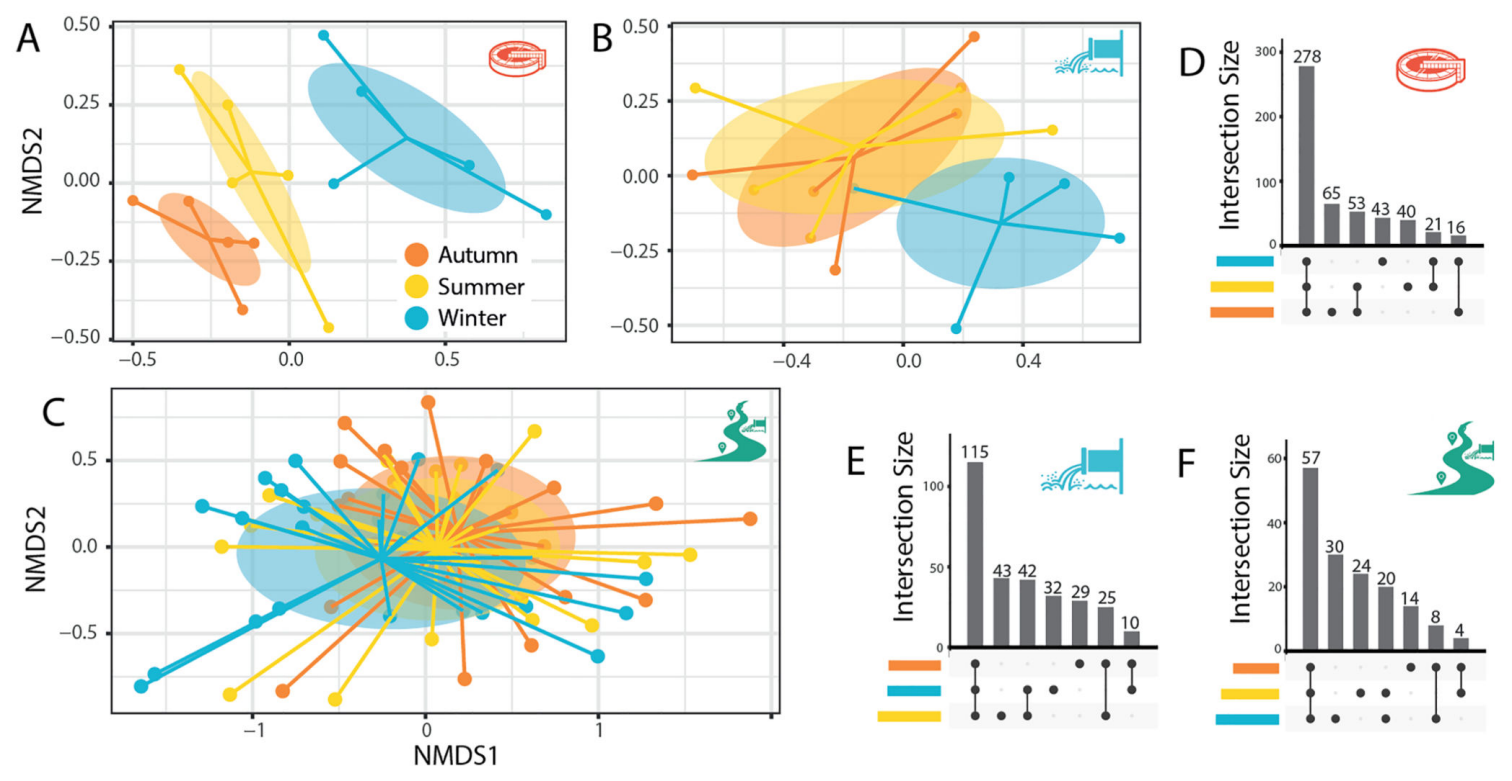
Non-metric multidimensional scaling (NMDS) plots show seasonal differences in the resistome of (A) untreated wastewater influent, (B) treated effluent, and (C) river sediment. UpSet plots show the number of shared ARGs among (D) untreated wastewater influent, (E) treated effluent, and (F) river sediment samples.

The seasonal relationships between resistomes in untreated waste-water, treated effluent and river sediments are shown in NMDS plots (Fig. 7A–C). Wastewater influent exhibited statistically significant differences in resistome profiles between seasons (Influent; Adonis; F_2,12_ = 9.82, R^2^ = 0.62, p ≤ 0.001) (Fig. 7A). However, this was not true for wastewater effluent, despite being collected on the same sampling dates as the influent (Adonis; F_2,12_ = 1.54, R^2^ = 0.20, *p* = 0.099) (Fig. 7B). Likewise, there was no evidence of seasonal differences in resistome profiles in river sediments (Adonis; F_2,72_ = 1.28, R^2^ = 0.03, *p* = 0.172) (Fig. 7C). There was no evidence of seasonal differences in ARG richness in the influent or effluent (*t*-test, *P* > 0.05). ARG richness was significantly higher in sediment samples in winter (mean = 227.8, SD = 37.77) than in autumn (mean = 273.2, SD = 23.28) (*t*-test; *P* = 0.0088) (Supplementary Fig. S5A–C). However, there was no evidence of differences in ARG abundance in influent, effluent, or sediment samples between seasons (Supplementary Fig. S5D–F).

Although there was a large shared resistome between seasons for influent, effluent, and sediment samples (ARGs found across all three seasons; influent *n* = 278, effluent *n* = 115, sediment *n* = 57), a smaller subset of ARGs were identified that were uniquely associated with individual seasons in all sample types (Fig. 7D–F), indicating some level of seasonal variation in ARG composition, even when this was not significant at the community level.

### 3.5 Associations with nutrients and organic pollutants

The input of wastewater changed the chemical composition of the five receiving rivers. Aggregated across sites and seasons, dissolved phosphorous, ammonium, chloride, nitrite, nitrate and organic carbon were significantly elevated in water samples taken downstream of the effluent source, up to 1000 m downstream (Supplementary Figs. S6 and S7). Across all samples, 113 different organic compounds were detected (Supplementary data S5), including antimicrobials (*n* = 21), herbicides (*n* = 20), analgesics (*n* = 15), pesticides (*n* = 10), fluorosurfactants (*n* = 7), mental health drugs (*n* = 7), anticonvulsants (*n* = 5), blood pressure drugs (*n* = 4), antihistamines (*n* = 3), sedatives (*n* = 3), and sweeteners (*n* = 3).

Of the antimicrobials measured here, only azithromycin, clarithromycin, metronidazole, sulfadiazine, sulfamethoxazole, sulfanilamide and trimethoprim, had Predicted No-Effect Concentrations (PNECs) for use in environmental risk assessment of antibiotics, based on the lowest value from collated by the AMR Industry Alliance of PNEC-ENVs (from Brandt et al. (2015) and Le Page et al. (2017) and PNEC-MIC values (from Bengtsson-Palme and Larsson (2016). Of these antimicrobials, azithromycin PNECs were most often exceeded in this study, with concentrations exceeding the lowest AMR Industry Alliance PNEC (PNEC-ENV = 0.03 μg/L) in four of five of the effluent samples and four of five of the downstream sediment samples, in comparison to one in five of the upstream water samples. The pattern in clarithromycin concentrations was similar, with measured concentrations meeting or exceeding the lowest AMR Industry Alliance PNEC (PNEC-MIC = 0.25 μg/L) in one in five effluent samples and two in five downstream water samples, compared to none in the upstream water samples. Metronida-zole concentrations only exceeded the PNEC (PNEC-MIC = 0.13 μg/mL) in one downstream sample. The other four antimicrobials measured here that are included in the AMR Industry Alliance list did not exceed the published PNECs (AMR Industry Alliance, 2023) (Supplementary Table 1).

Although the azithromycin PNEC was most exceeded in downstream sediment and effluent samples, the highest RQ (i.e., highest concentration and exceedance of PNEC) was found in an upstream water sample (RQ = 11.3). For clarithromycin and metronidazole, RQs above 1 were found in downstream water (clarithromycin RQ=1.36; metronidazole RQ = 1.15) and effluent samples (clarithromycin RQ = 1.88; Supplementary Table 1).

## 4 Discussion

WwTWs are critical in maintaining public health by reducing human and environmental exposure to untreated wastewater. The purpose of this study was to understand better the role of this engineered human-environment interface in the mitigation and environmental dissemination of AMR. We used metagenomics to reveal the composition, abundance, and richness of ARGs, ISs, and Enterobacterales plasmids in untreated and treated wastewater and in sediments from effluent receiving rivers and aimed to identify generalisable patterns in the transformation and dissemination of AMR along this continuum.

Our results are supported by prior research on AMR in wastewater. For example, similar compositional changes in the antimicrobial resistome during wastewater treatment were observed by Dai et al. (2022), who recorded a significant compositional shift in ARGs from influent to activated sludge, and Li et al. (2021), who observed reductions in 70 clinically important extended-spectrum β-lactamase and carbapenemase genes during treatment. We also observed reductions in the abundance and richness of ARGs, ISs, and plasmids during treatment. Similar trends have been observed. For example, Ping et al. (2022) reported an overall removal efficiency of 65.6 % for all ARGs from influent to effluent in eight WwTWs in China.

Reductions in AMR occurred during wastewater treatment despite the high levels of antimicrobial chemicals reported in wastewater (Read et al., 2022). These antimicrobials might be expected to maintain or even increase AMR during transit through wastewater systems, as they are frequently measured at concentrations above those predicted to select for resistance (Bengtsson-Palme and Larsson, 2016). The most likely mechanisms for the changes that we and other researchers have observed are the ecological drivers of microbial communities as they pass through and reside in different stages of the wastewater treatment process. Despite the different secondary treatment processes and the presence/absence of tertiary treatment at the five WwTWs, we observed consistently large shifts in the ARG, IS, and Enterobacterales plasmid compositions between the influent and effluent. One possible explanation is that changes in bacterial and archaeal community composition between influent and effluent, as has been observed previously (Numberger et al., 2019), driven by the ecological niches that exist in the biological treatment process, override AMR selection processes, resulting in AMR removal during treatment. Additionally, PNEC thresholds are primarily determined using laboratory-based single--species assays (Roos et al., 2012). Therefore, these thresholds might not be appropriate for chemically rich and microbiologically diverse wastewater environments. More work is needed to understand how the mechanisms by which the wastewater treatment environment transforms AMR and the potential for ARG co-selection due to exposure to other chemicals.

Despite reductions in AMR in treated effluents, we observed significant impacts of wastewater effluents, with sediments downstream containing a higher richness and normalised abundance of ARGs, ISs, and Enterobacterales plasmids to at least 1000 m downstream (the limit of our sampling). However, there was a decline in ARG richness between 100 m and 1000 m downstream, indicating reduced impact further downstream from the wastewater source. Previous research has found similar trends, with water and sediment in rivers, streams, and lakes downstream of wastewater effluent sources having elevated abundance and richness of ARGs (Quintela-Baluja et al., 2019), but also that the microbial community and resistome structure at least partially recovers from the effluent impact with increasing distance from the source (Price et al., 2018). One possible mechanism is AMR-carrying microbes attached to particles found in the effluent (Yu et al., 2020) settle into river sediments. It is important to note that various ARGs were present in the relatively unimpacted upstream environments, although at low abundances. Differential abundance analysis identified only one ARG (23S rRNA with mutation conferring resistance to macrolide antibiotics) as consistently more abundant at upstream locations. Macrolides are natural products of secondary metabolism in many actinomycetes (Al-Fadhli et al., 2022), and such ARGs are present in a wide range of environmental microbes that are not associated with human activity (Paun et al., 2021). However, ARGs were much more abundant and richer in wastewater influent and effluent than in river sediments upstream of the effluent source. As a result of wastewater exposure, ARGs were 543 % more abundant and 163 % higher in richness in downstream sediments than upstream sediments, highlighting the role of effluent as a significant source of AMR in the freshwater environment.

Analysis of the contribution of wastewater to the sediment resistome using FEAST showed that the proportion of ARGs that could be assigned to wastewater increased from 0.1 in upstream sediments to between 0.31 and 0.39 in downstream sediments. This did not decrease along the downstream transect, showing that the impact of effluent on elevating ARGs in rivers extends to at least 1000 m downstream (the limit of our study), but likely much further within the river network. The fact that there was an influent signal in the upstream sediment resistome may reflect the fact that a proportion of the influent ARGs originate from environmental sources, as much of the UK sewerage network is combined and takes water from drainage and road runoff as well as sewage. Additionally, under low-flow conditions, wastewater may flow upstream in the river channel, or there could be additional, undocumented sources of AMR-elevating pollution in the upstream catchment, such as agricultural or urban drainage sources, elevating upstream levels of AMR. Catchment-scale studies on the distribution of AMR are uncommon. However, Amos et al. (2015) found evidence that WwTWs accounted for 49.5 % of the variance in resistance levels across the Thames catchment, UK, and Elder et al. (2021) identified wastewater emissions as the main driver of antibiotic and antibiotic resistance gene presence in the Avon catchment, UK.

We observed significant seasonal differences in the beta diversity of AMR in wastewater influent but not in wastewater effluent or sediments. However, we did not observe seasonal differences in the richness or normalised abundance of ARGs. Seasonal variations in the consumption of antibiotics have been widely reported for both the UK and other European countries, with peaks observed in winter months due to prescribing for respiratory tract infections (Ferech et al., 2006). As well as respiratory infections, which tend to peak in the winter months, seasonal patterns have been observed in urinary tract infections (Rosello et al., 2017) and in AMR rates in community-acquired *E. coli* bloodstream infections, potentially driving both the prescribing of antimicrobials and the shedding of resistant microbes and genes into the wastewater network. However, seasonality in AMR in wastewater is less well studied. Seasonal fluctuations in antimicrobials that correspond with prescribing rates have been observed in wastewater influents (Coutu et al., 2013), and Comber et al. found that the performance of wastewater treatment processes improved under warmer conditions, leading to lower concentrations of antimicrobials in effluents in autumn when surface water/sewage treatment temperatures tend to peak (Comber et al., 2020). Our results indicate that despite seasonal variation in the inputs of AMR into treatment works, the treatment processes, as well as restructuring the bacterial and archeal community and resistome, appear to act as a seasonal homogeniser, resulting in less pronounced differences in AMR emissions.

Previous research has identified seasonal variation in specific ARGs measured in river water samples. For example, Keen *et al*. identified fluctuations in normalised loads of *tet* genes that were linked to river flow (Keen et al., 2018), and Rieke *et al*. showed that higher resistance gene concentrations in artificial drainage samples occurring in spring and autumn were likely linked to agricultural manure applications (Rieke et al., 2018). The lack of strong seasonal variation in AMR in sediments observed in this study may result from the lack of seasonality in AMR in wastewater effluents. In addition, river sediment and biofilm bacterial and archeal communities are under higher levels of deterministic ecological selection (Gweon et al., 2021), which may provide resilience to seasonal variations in AMR.

We found that the normalised abundance of the human gut-associated bacteriophage crAssphage was strongly associated with the normalised abundance and richness of ARGs. This is consistent with other studies showing that crAssphage can indicate human faecal contamination in surface and groundwaters (Sabar et al., 2022). Additionally, Karkman *et al*. showed that the normalised abundance of crAssphage was positively correlated with the normalised abundance of AMR across a wide range of environments and countries. The strong positive association observed in this study further highlights the utility of crAssphage as a marker for human faecal pollution in freshwater environments and its strong association with AMR.

In addition to modifying the genetic composition of river sediments, the release of treated wastewater causes changes in the chemical composition of the river environment. We observed elevated levels of nutrients such as different forms of dissolved and total phosphorus, nitrogen (nitrite, nitrate and total nitrogen) and dissolved organic carbon. Downstream water samples also had a higher richness and total loads of organic chemicals measured by LC-MS, including known antimicrobials. A limitation of observational studies such as this one is that it is challenging to disentangle the true cause of elevated AMR and AMR-related genetic markers in wastewater-impacted environments. Wastewater introduces viable and dead microbes (some of which contain resistance mechanisms), extracellular DNA that contains ARGs, nutrients that support the growth of microbes, and antimicrobial compounds that may inhibit growth and select for resistance in microbes native to the freshwater environment. Disentangling the relative contributions of each of these factors to the elevated levels of AMR observed in this study, and others, will require experimental approaches, examining each of these factors individually and in combination, in receiving environments that represent different freshwaters (e.g., water chemistries, ecologies, flows and levels of dilution).

Finally, although this study encompassed five different wastewater treatment works (including three activated sludge and two trickling filter processes) and receiving environments, we were not able to draw any significant conclusions about the relative role of different treatment mechanisms in reducing or modifying the transmission of AMR. The number and size of surveys that study AMR in wastewater is increasing, but wastewater treatment processes in the UK represent a huge range of treatment processes and configurations, serving populations of different sizes with different demographics, prescribing rates, and industrial inputs. As a result, there is a need for large, well-replicated studies of AMR entering and leaving WwTWs across a wide range of treatment processes and wastewater catchments to generate data on the scale needed to identify the role that different treatment processes play.

## 5 Conclusions

Our study performed high-depth metagenomics (approximately 80 M reads per sample) to examine the transformations in the antimicrobial resistome between untreated wastewater (influent), treated wastewater effluent and upstream and downstream river environments at five wastewater treatment works in Oxfordshire, UK. We observed significant shifts in the wastewater resistome and sequence reads matching AMR-associated mobility (insertion sequences and *Enterobacteriaceae* plasmids) when it underwent treatment, with an 87.4 % reduction in ARG abundance and a 63.4 % reduction in ARG richness. The addition of wastewater to receiving environments caused an increase in both ARG abundance and richness in sediments, highlighting the impact that wastewater has on AMR levels in freshwaters. We also observed that elevated levels of AMR in river sediments persisted to at least 1000 m downstream of the effluent entry point, illustrating how the impacts of wastewater can be disseminated further across river networks. This highlights three important areas for future research. The first is to develop tools to predict AMR dissemination within river networks, allowing models to be developed that can be scaled up to whole river catchments. The second is to understand better the potential risks associated with elevated AMR in freshwaters. This includes understanding the potential threat to the use of freshwater for clean and safe drinking water, as well as to recreational users of freshwater environments. The final is to characterise which treatment processes could be most effective at potentially decreasing the environmental dissemination of AMR from wastewater.

## Supplementary Material

Supplementary methods and figures

Supplementary tables/data

## Figures and Tables

**Table 1:** The five wastewater treatment works (WwTWs) sampled in this study, including their Population Equivalent (PE), primary, secondary, and (where present) tertiary treatment processes (PST = Primary Settlement Tanks; ASP = Activated Sludge Process; Filters = trickling filter beds), consented flow (m^3^/day), location (latitude and longitude), and name of the effluent receiving river.

WwTW	Population equivalent (PE)	Primary treatment	Secondary treatment	Tertiary treatment	Consented Flow (m^3^/d)	Lat, long	Effluent receiving river
Oxford	223,435	PSTs	ASP	N/A	50,985	51.71358, −1.21439	Littlemore brook to River Thames
Witney	49,522	PSTs	ASP	Disc filters	11,883	51.77307, −1.49787	Collwell Brook to River Windrush
Didcot	37,731	PSTs	ASP	Sand filters	11,476	51.61775, −1.25057	Moor Ditch to River Thames
Wantage	26,905	PSTs	Filters	N/A	6250	51.6205, −1.42043	Letcombe Brook to River Ock
Watlington	2841	PSTs	Filters	N/A	2000	51.65265, −1.02441	Pyrton stream to River Thame

## Data Availability

The datasets supporting the conclusions of this article are included within the article and its additional files. All raw sequence data are available from the EBI European Nucleotide Archive, project PRJEB34634. Water chemistry data can be found at https://doi.org/10.5285/80710d5e-06cf-4757-93c5-87fcbe421352. Processed AMR data used for this study are found in the supplementary data.

## Data availability

Raw data in public repositories, processed data attached as supplementary.
