# Supplementary material for: Dissemination and persistence of antimicrobial resistance (AMR) along the wastewater-river continuum
Source: Water Res. Author manuscript; Available in PMC 2025 Mar 10. (PMC7617467; doi:10.1016/j.watres.2024.122204)
Supplement: Supplementary methods and figures [file EMS203363-supplement-Supplementary_methods_and_figures.docx]

Supplementary methods.

**Nutrient chemistry.**

The bulk water samples were immediately subsampled into 60mL bottles for total phosphorus (TP) analysis. Other subsamples were filtered immediately in the field through a 0.45µm cellulose nitrate (Whatman WCN grade) membrane filter into 60mL bottles, for dissolved nutrient and major anion analyses. All bottles were acid-washed and rinsed in deionised water before use. The water samples were stored at 4°C in the dark until they were returned to the laboratory (within 6h). Water and effluent samples were analysed for a range of physicochemical parameters, including soluble reactive phosphorus (SRP), total dissolved phosphorus (TDP), total phosphorous (TP), dissolved ammonium, dissolved nitrite, dissolved nitrate, total dissolved nitrogen (TDN), dissolved fluoride, dissolved chloride, dissolved sulphate, and dissolved organic carbon, using methods described in detail previously (Bowes *et al*., 2018, 2012).

**Sample processing and DNA extraction.** Sewage influent samples were processed by centrifuging 450mL of influent per sampling event in x9 50mL centrifuge tubes at 6000 g for 20 min at 4°C. The supernatant was discarded, and the pellets were resuspended in 5mL of molecular-grade water. The pellets were then pooled into a single 50mL tube, centrifuged again at 6000g for 20 min at 4°C and the supernatant was discarded. The final composite pellet was stored at -20°C until DNA extraction. Up to 500mL of each effluent sample (n=15) was filtered through a 0.22μm pore size Durapore membrane filter (Merck, UK), and the filters were stored at -20°C until DNA extraction. DNA extraction was carried out on effluent filter membranes, or 200μg subsamples of influent biomass using the Qiagen PowerSoil kit (Qiagen, UK), following the manufacturer’s instructions.

Sediment samples from each site were thawed at room temperature before manual homogenisation by shaking and sieving using a 2 mm sterile sieve. A 0.2 g subsample was removed using a sterile single-use spatula, and DNA extraction was carried out using the Qiagen PowerSoil kit (Qiagen, UK). After DNA extraction, near-, mid-, and far-bank DNA samples from each river location were pooled before metagenomic sequencing.

**DNA sequence processing and metagenomic normalisation**

For quality filtering, we used a length cut-off of 75 bp and average Phred score ≥ 25, and the first 13 bp of Illumina standard adapters (AGATCGGAAGAGC) for adapter trimming.

The relative abundance of reads mapping to genes was multiplied by 1,000,000 to give a “Fragment/Reads Per Million” (FPM). To account for the variation in AMR gene lengths, the FPMs of each AMR gene were divided by its length in kb resulting in FPKM. Finally, we used the per-sample abundance of 31 single-copy genes to normalise the FPKM values, dividing the FPKM value by the average count for the 31 single-copy genes to yield a proxy of “AMR genes per bacterial cell”.

Tables containing AMR sequence variants, ISs, Enterobacterales plasmids, and bacterial taxonomy data were normalised to the average per-sample count of 31 single-copy genes to adjust for variable sequencing depth per sample and provide a ‘community prevalence’. We chose this as the optimal normalisation method, as influent, effluent, and sediment samples represented very different sample types in terms of mass/volumes analysed, as well as containing large variations in bacterial biomass. Normalising to single-copy genes provides a measure of ARG prevalence within the community, regardless of microbial density across the different sample matrices (Davis *et al*., 2023).

To increase confidence in the presence or absence of AMR genes, a sample was considered to contain an AMR gene if the FPKM value was >0.1. Individual AMR genes were then grouped by inferred resistance to antibiotic classes or class gene families.

**References**

Bowes, M.J., Armstrong, L.K., Harman, S.A., Wickham, H.D., Nicholls, D.J.E., Scarlett, P.M., Roberts, C., Jarvie, H.P., Old, G.H., Gozzard, E., Bachiller-Jareno, N., Read, D.S., 2018. Weekly water quality monitoring data for the River Thames (UK) and its major tributaries (2009–2013): the Thames Initiative research platform. Earth Syst Sci Data 10, 1637–1653. <https://doi.org/10.5194/essd-10-1637-2018>

Bowes, M.J., Gozzard, E., Johnson, A.C., Scarlett, P.M., Roberts, C., Read, D.S., Armstrong, L.K., Harman, S.A., Wickham, H.D., 2012. Spatial and temporal changes in chlorophyll-a concentrations in the River Thames basin, UK: Are phosphorus concentrations beginning to limit phytoplankton biomass? Sci Total Environ 426, 45–55. <https://doi.org/10.1016/j.scitotenv.2012.02.056>

**Supplementary Figures 1A – 1E.** Maps showing the locations of the five sampling sites, and the sampling locations at each site upstream and downstream of the wastewater treatment works (WwTWs).


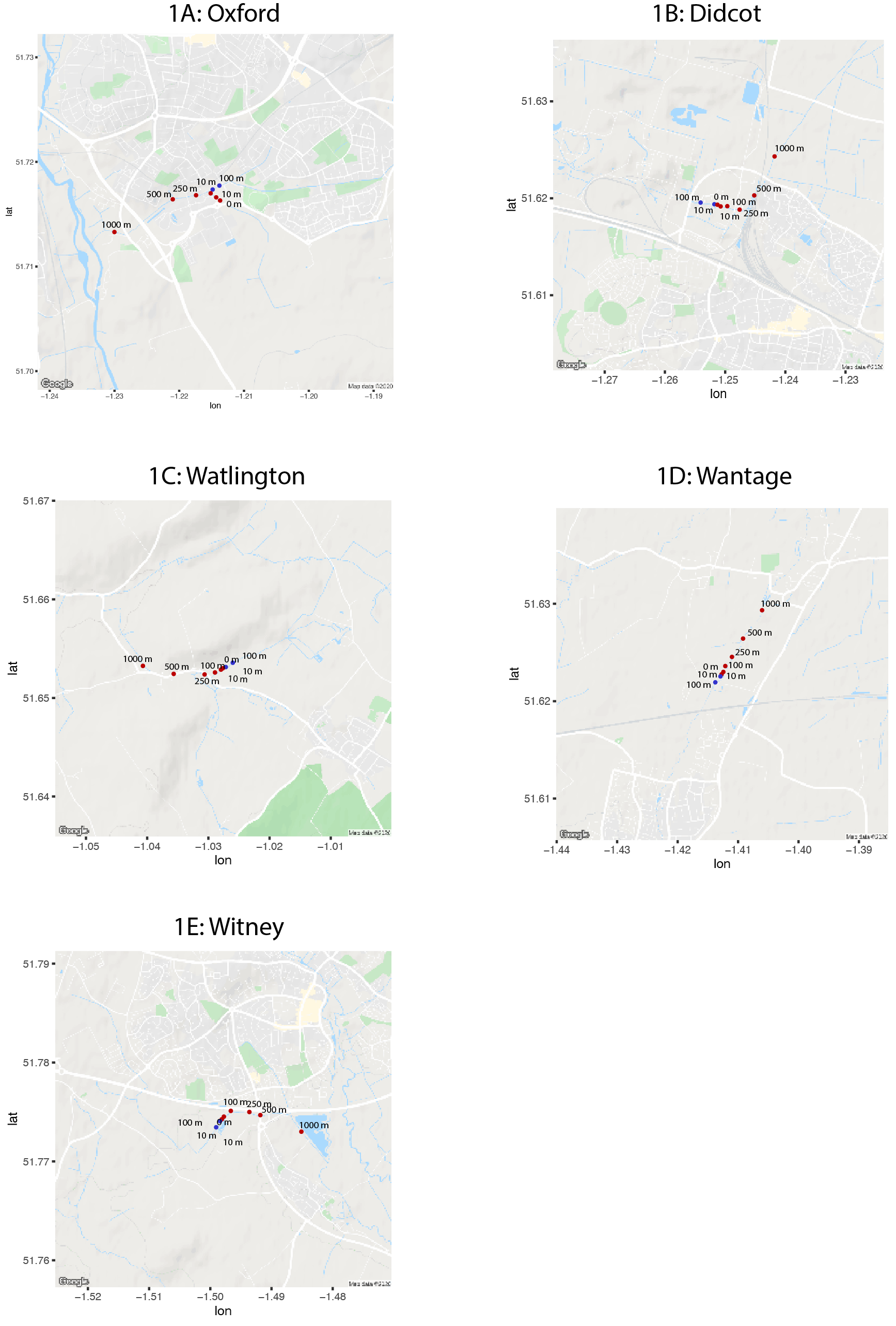


**Supplementary Figure 2A.** Boxplot showing the differences in the log richness of antimicrobial resistance genes (ARGs) across all five sampling locations at the upstream (-100 m), and downstream (100 m, 250m, 500m and 1000m) sites. The table shows the significance of a Student's t-test for each downstream site against the 100m upstream site. **** = P < 0.001.


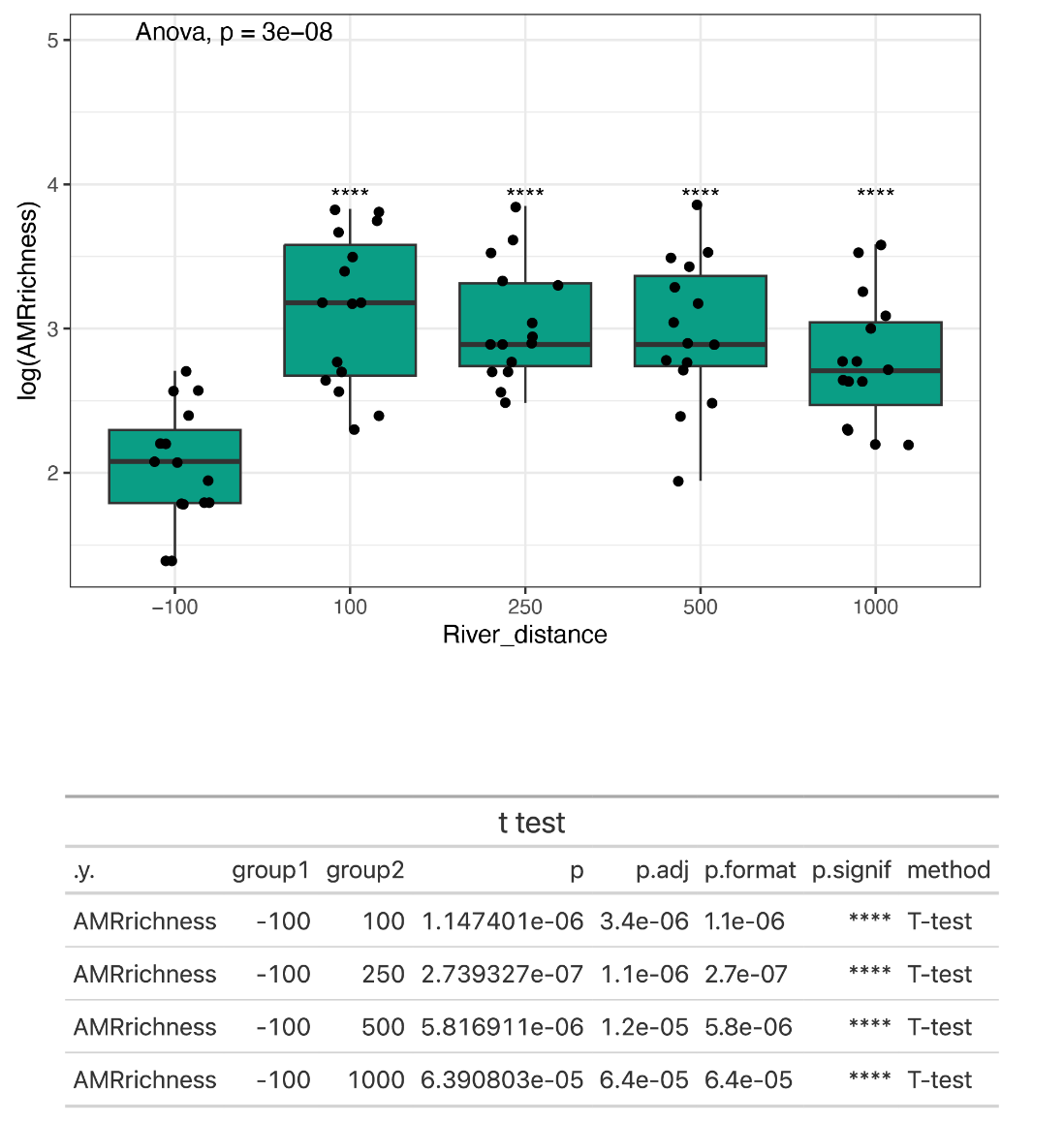


**Supplementary Figure 2B.** Boxplot showing the differences in the log normalised abundance of antimicrobial resistance genes (ARGs) across all five sampling locations at the upstream (-100 m), and downstream (100 m, 250m, 500m and 1000m) sites. The table shows a Student's t-test for each group against the 100m upstream site. **** = P < 0.001.


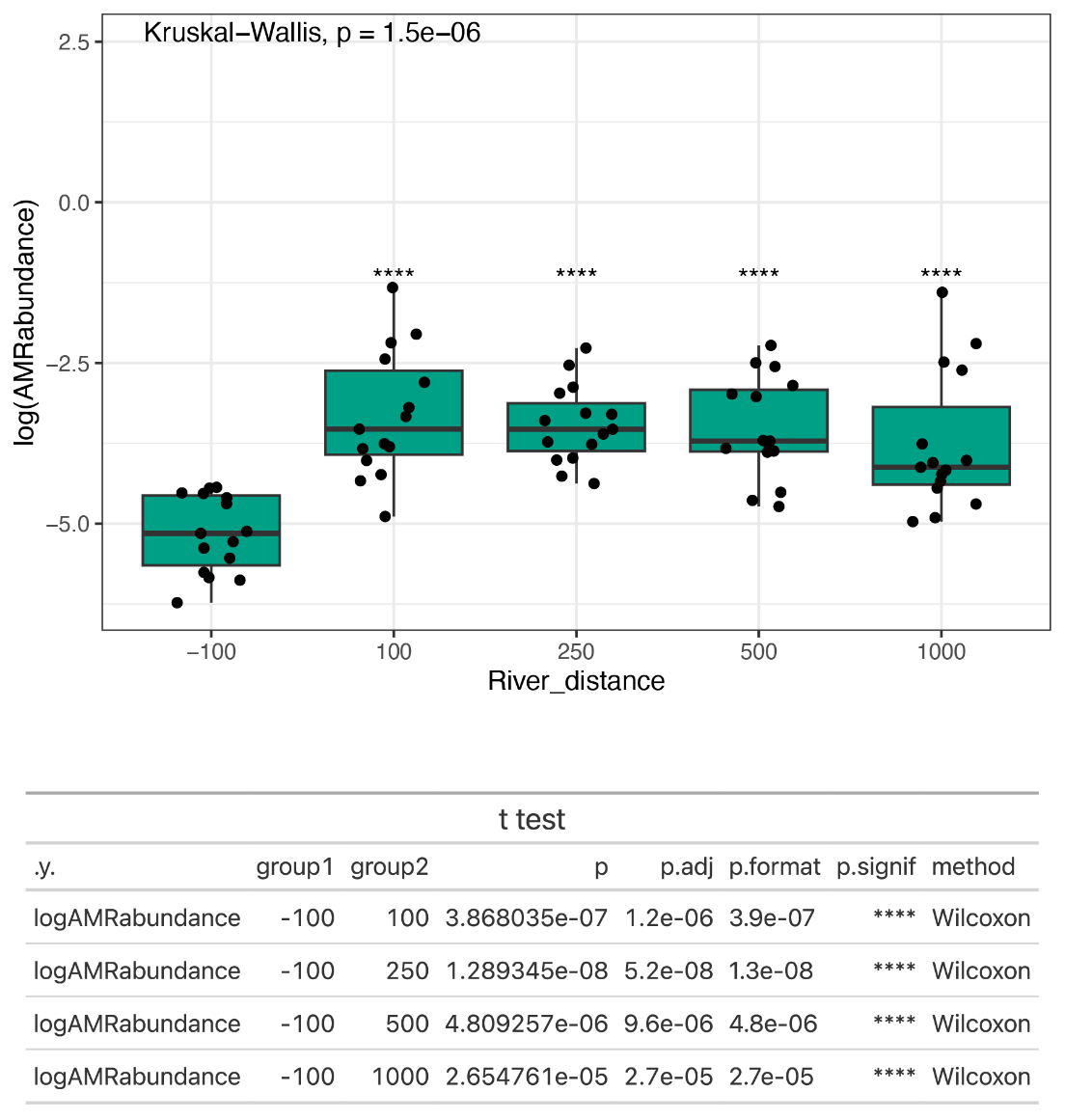


**Supplementary Figure 3.** Linear regression of the distance downstream of the wastewater treatment works (in metres) against log AMR richness.


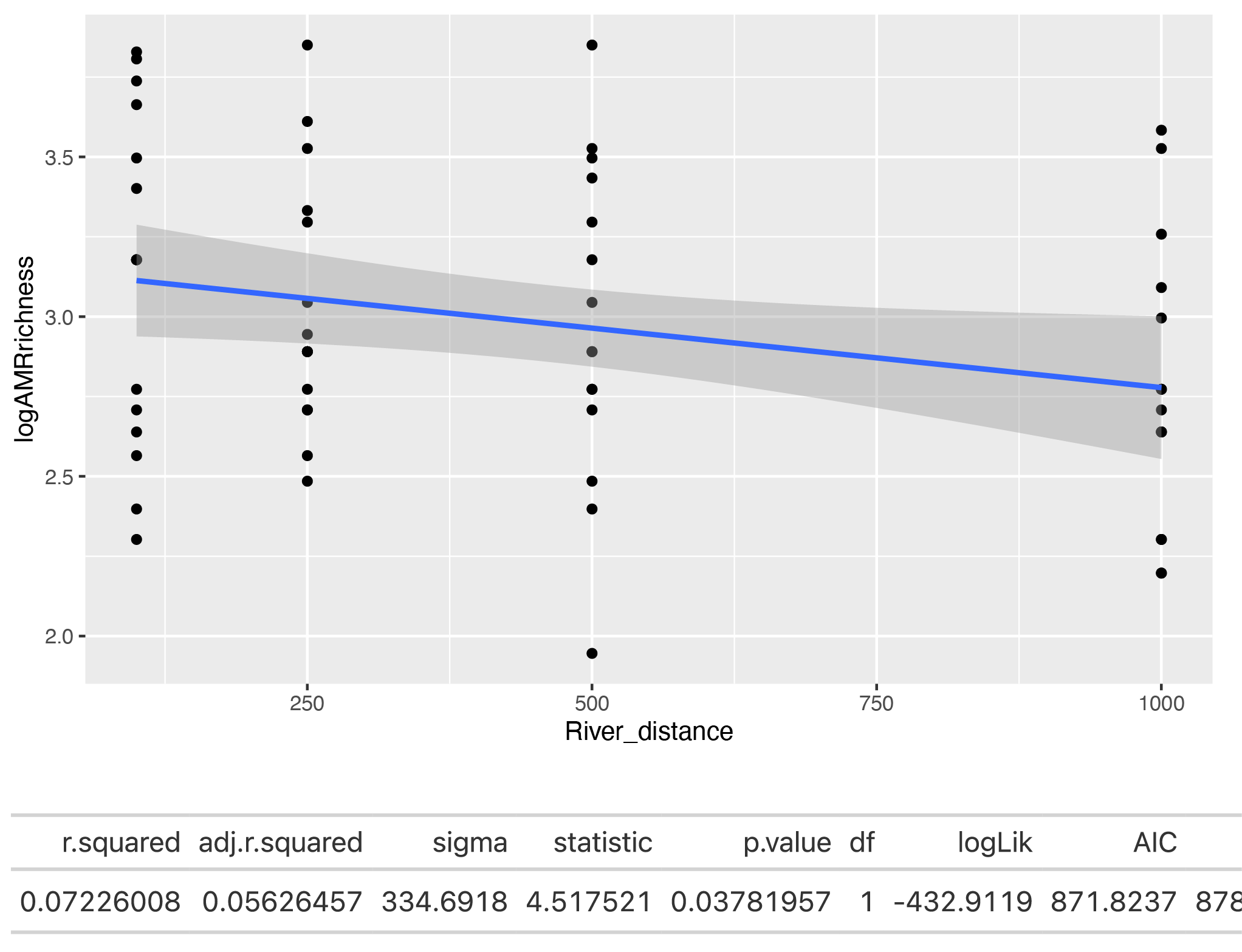


**Supplementary Figure 4.** Non-Metric Multidimensional (NMDS) plots showing differences in ARG composition at each of the five sampling locations (Oxford, Didcot, Wantage, Watlington and Witney). Tables with each plot show the results of Permutational Multivariate Analysis of Variance using Adonis from the Vegan R package.


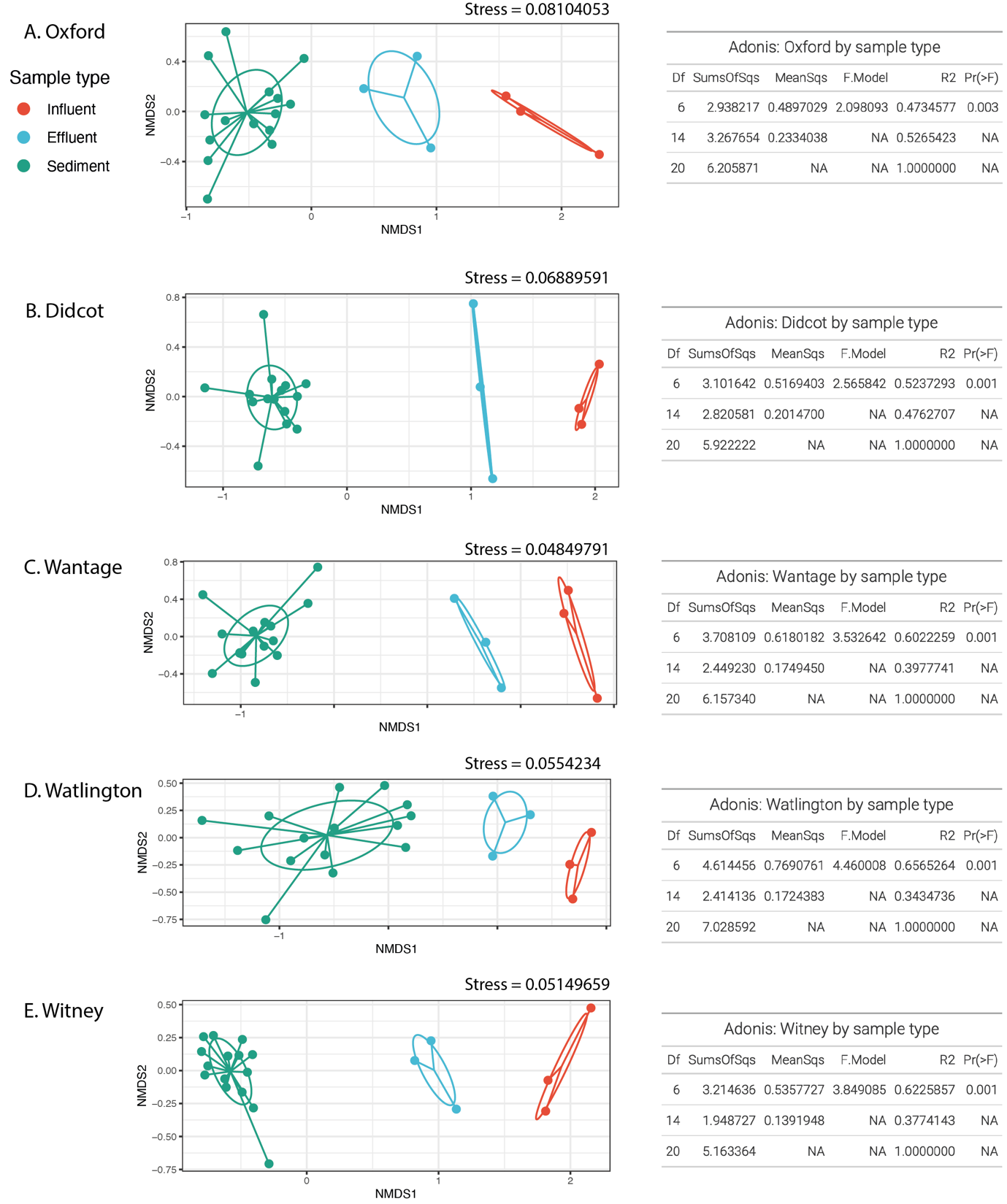


**Supplementary Figure 5.** Comparison of the richness of antimicrobial resistance genes (ARGs) from; (5A) influent, (5B) effluent, and (5C) sediment samples across all five sampling locations. Comparison of the normalised abundance of antimicrobial resistance genes (ARGs) from; (5D) influent, (5E) effluent, and (5F) sediment samples across all five sampling locations.

**Supplementary 5A**. Influent

**
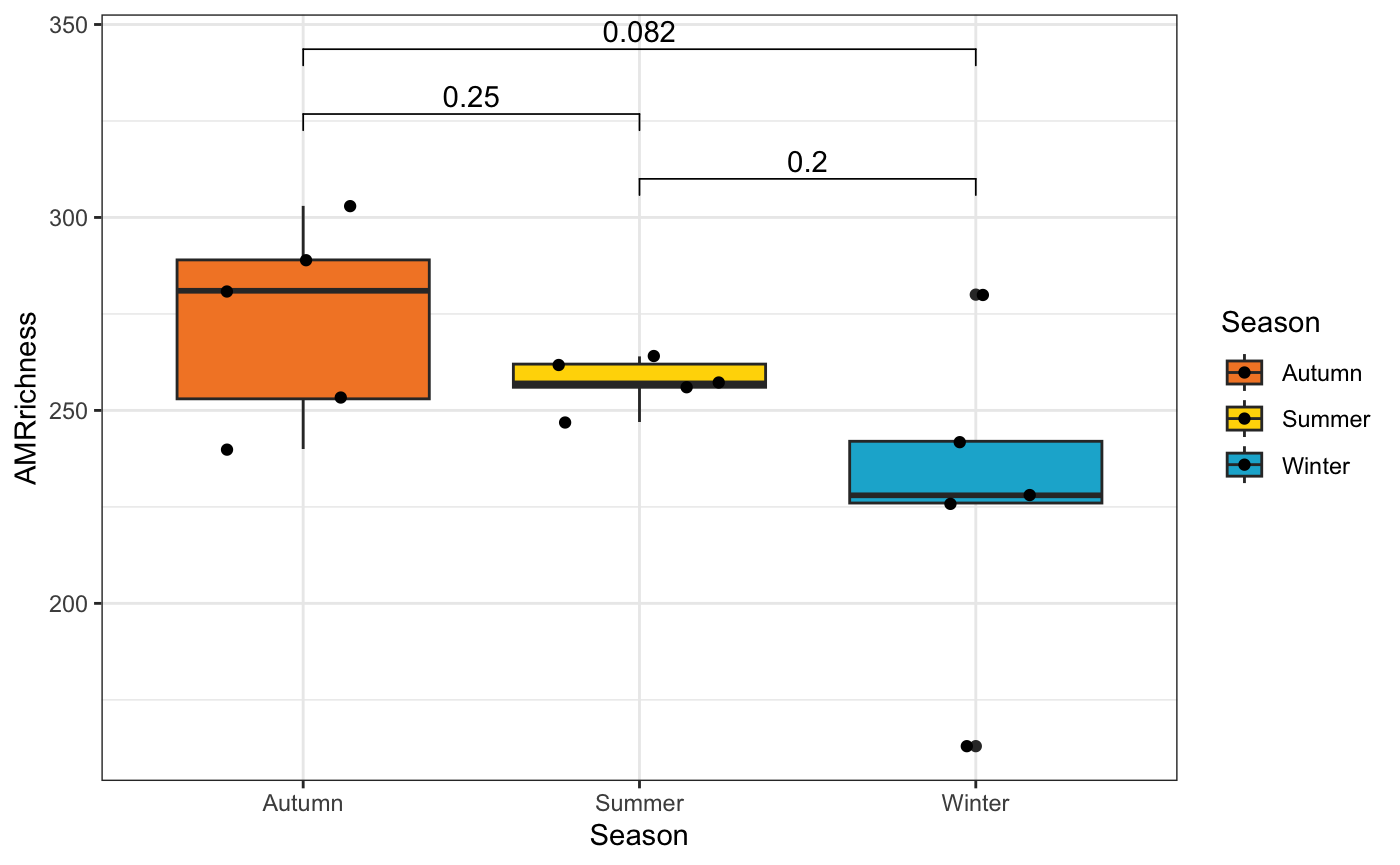
**

**Supplementary 5B**. Effluent


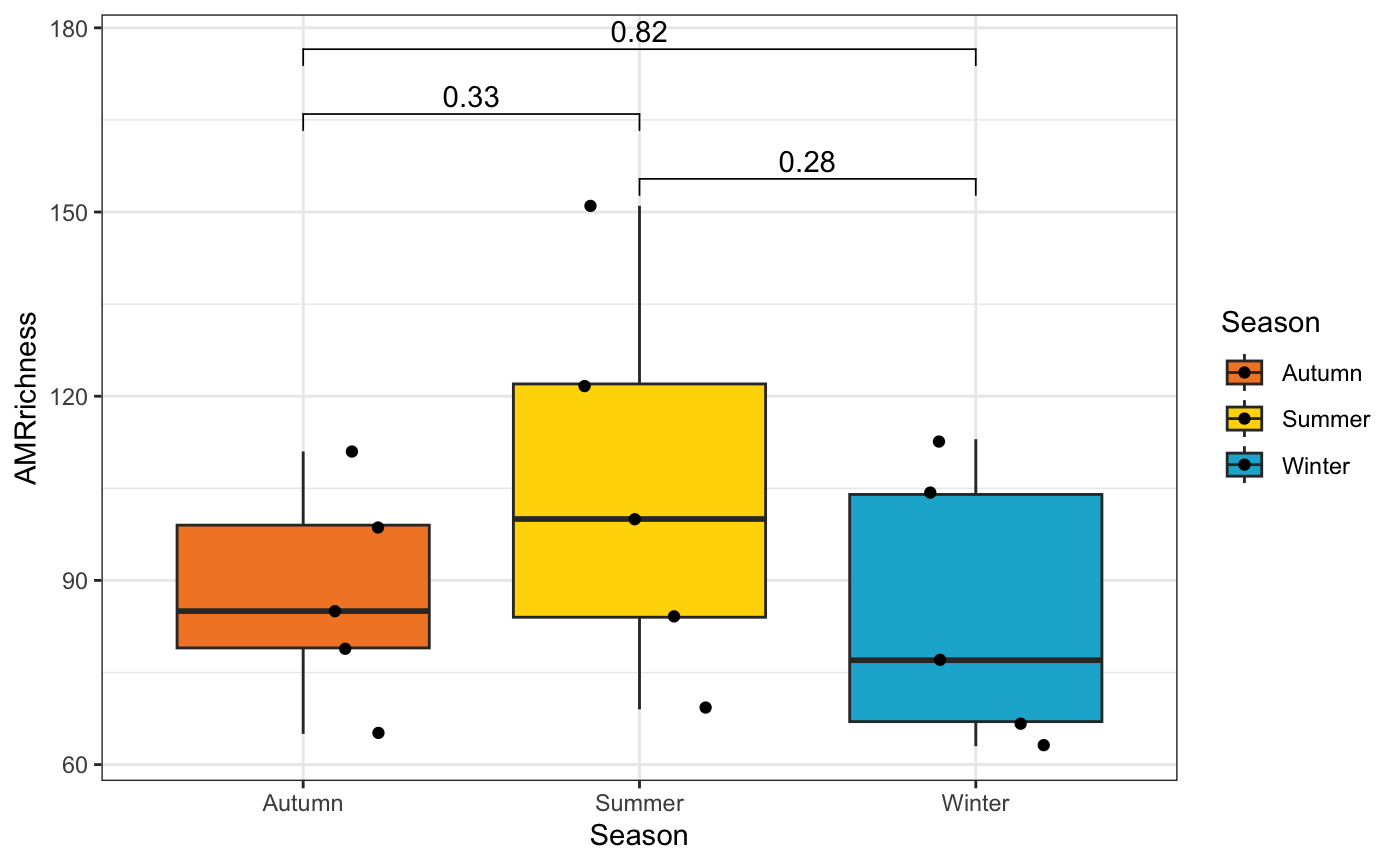


**Supplementary 5C**. Sediment


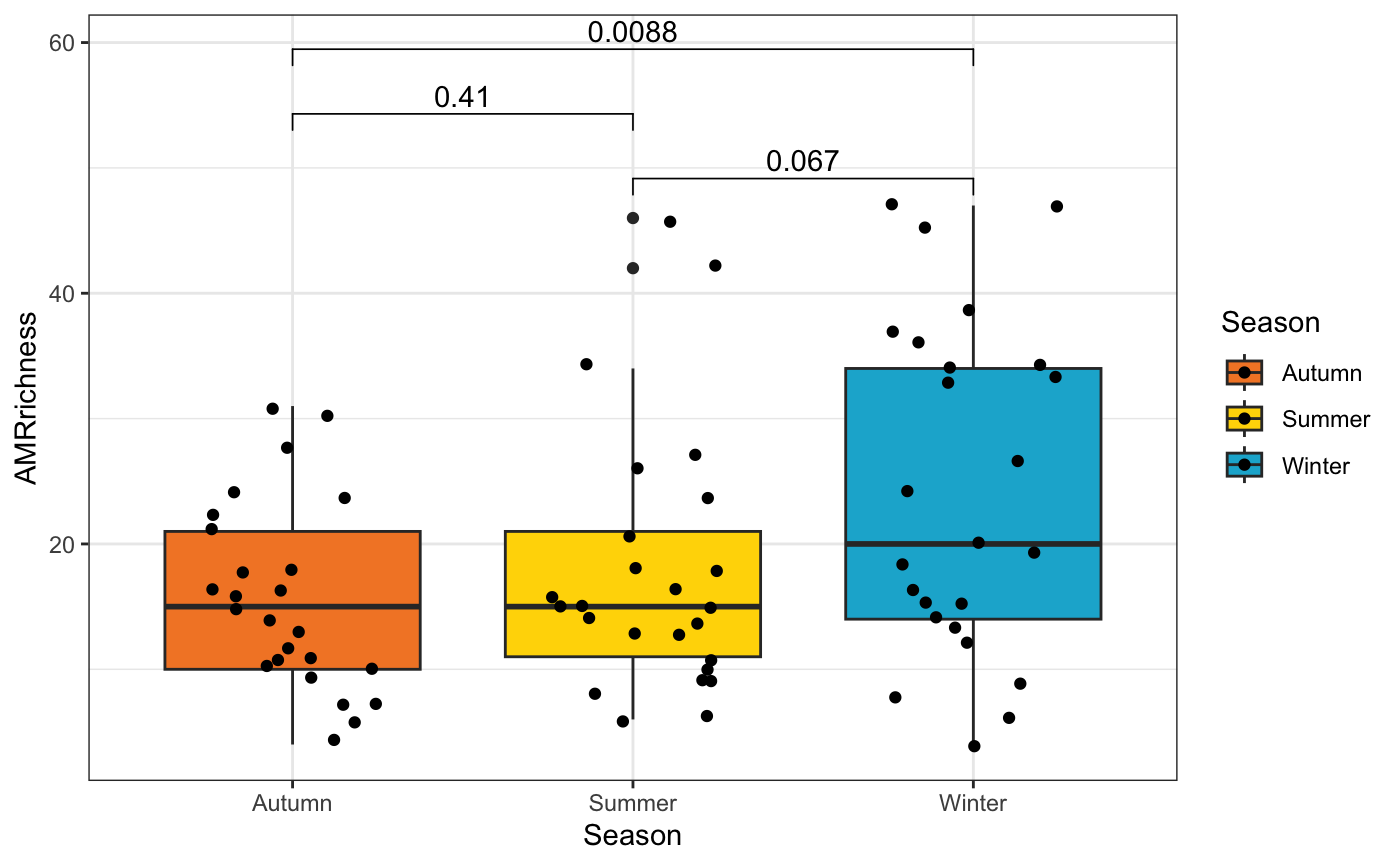


**Supplementary 5D**. Influent


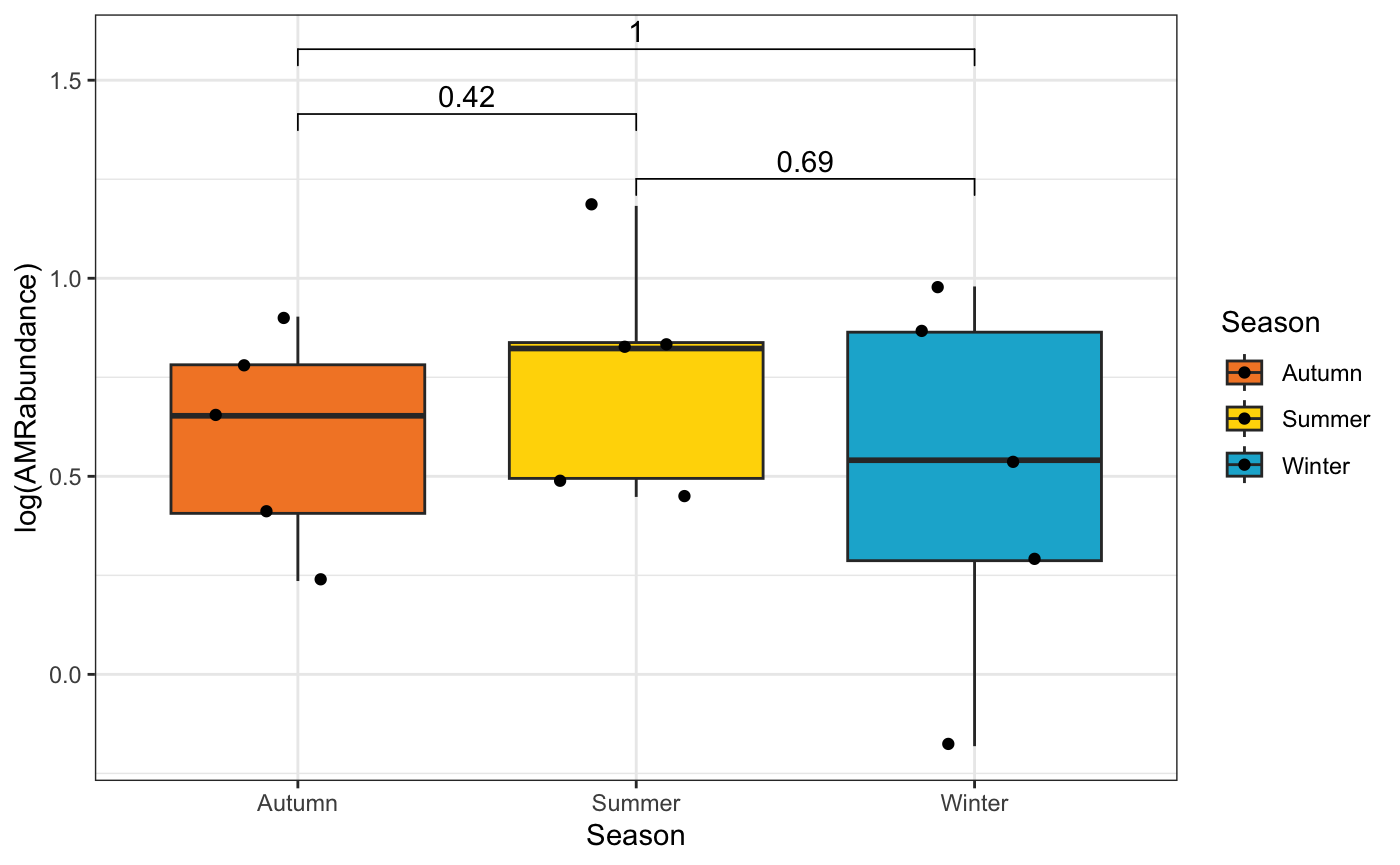


**Supplementary 5E**. Effluent


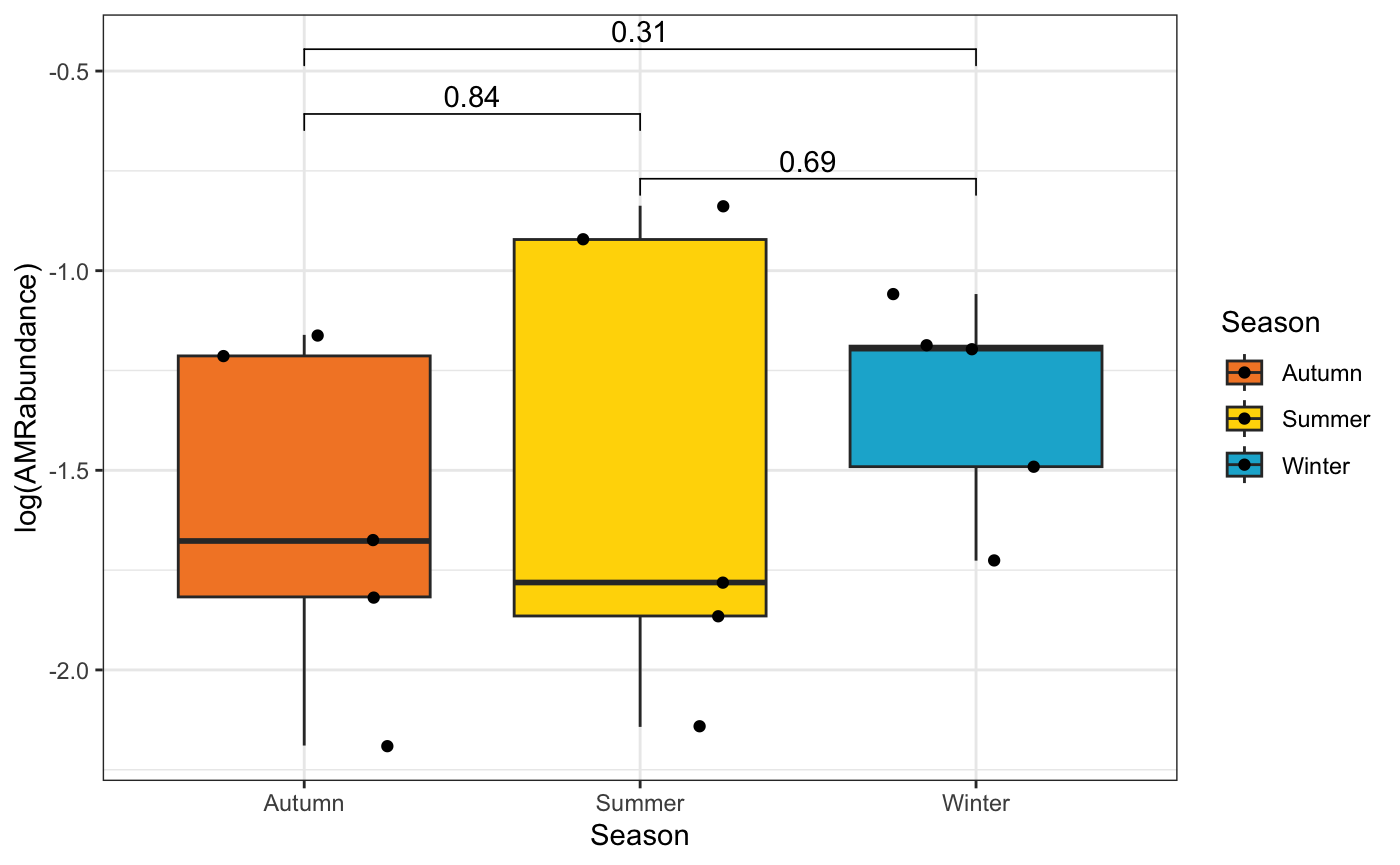


**Supplementary 5F**. Sediment

**
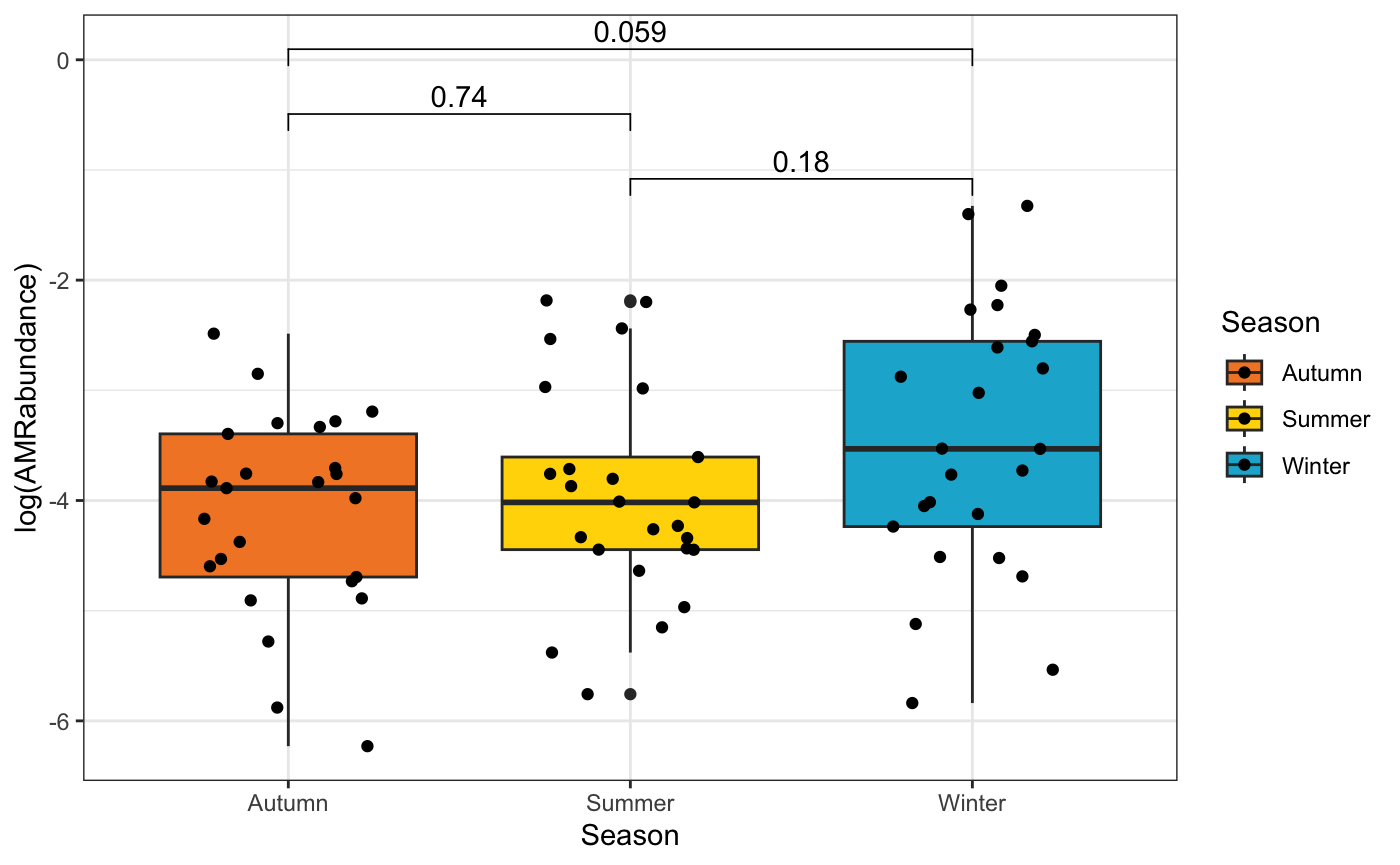
**

**Supplementary Figures 6.** Plots showing measured nutrients from rivers at each of the five sampling locations, from 100 m upstream to 1,000 m downstream.

**Supplementary Figures 6A.** Total dissolved phosphorus (µg/L).

**
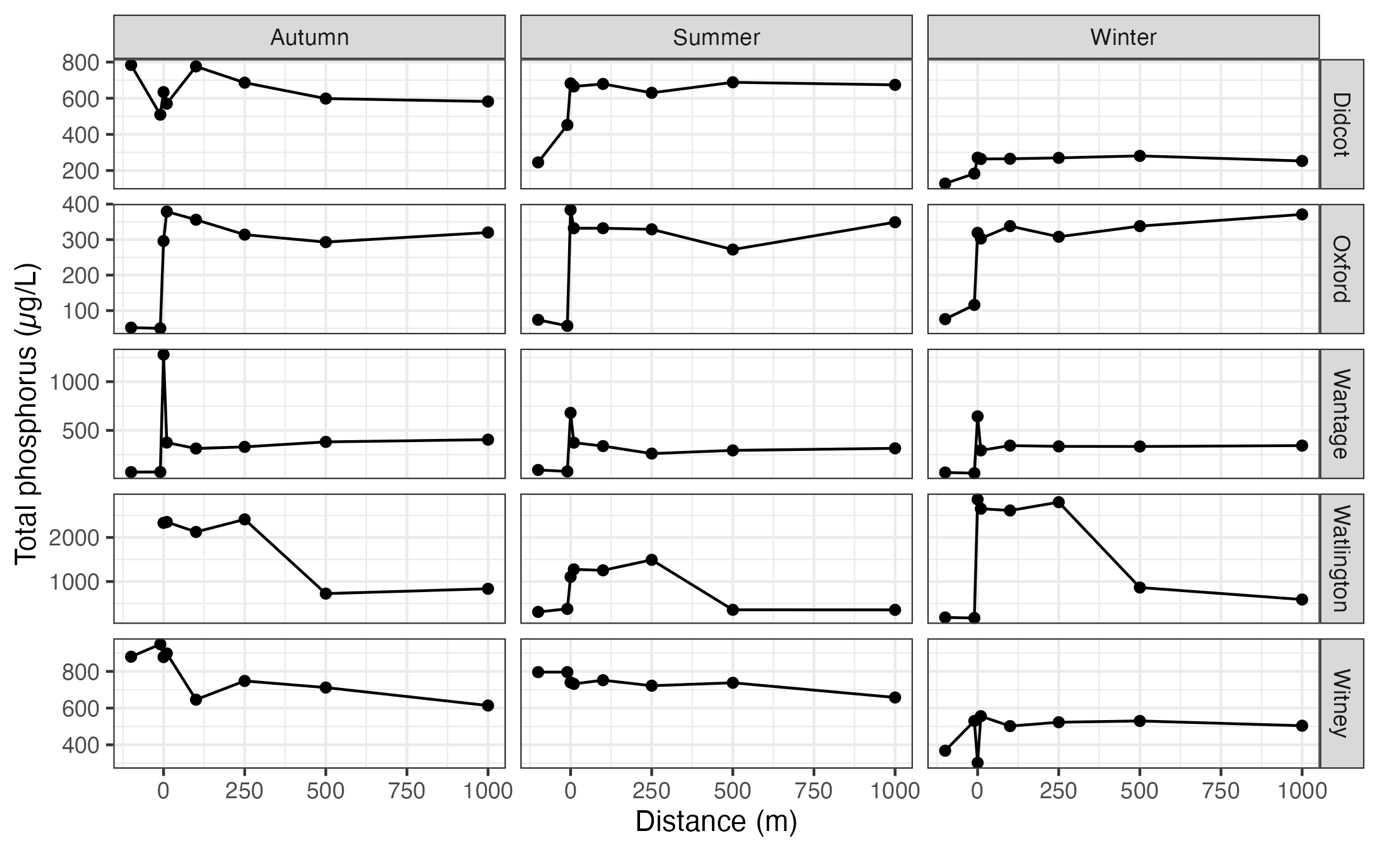
**

**Supplementary Figures 6B.** Dissolved organic carbon (mg/L).


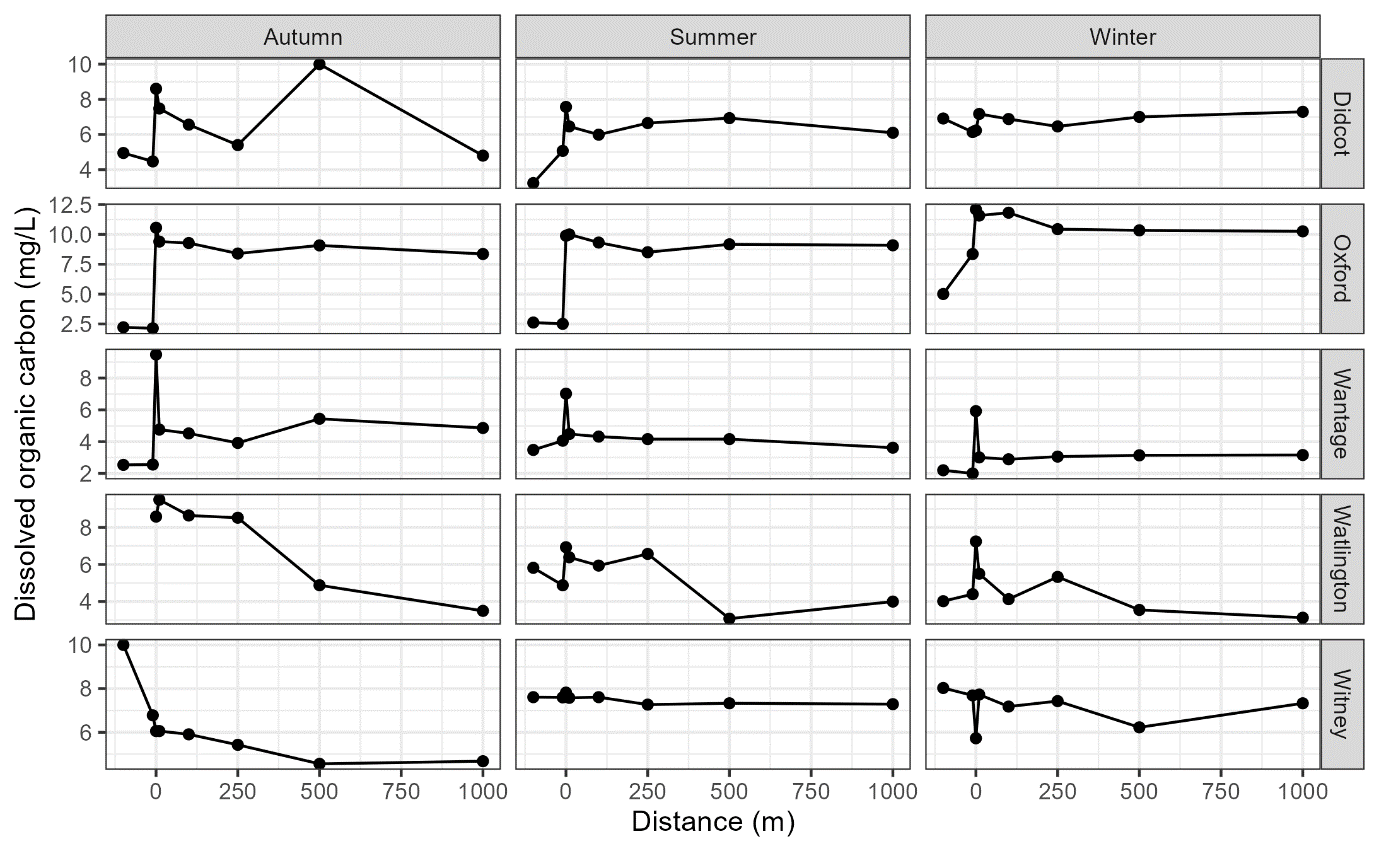


**Supplementary Figures 6C.** Total dissolved nitrogen (mg N/L).


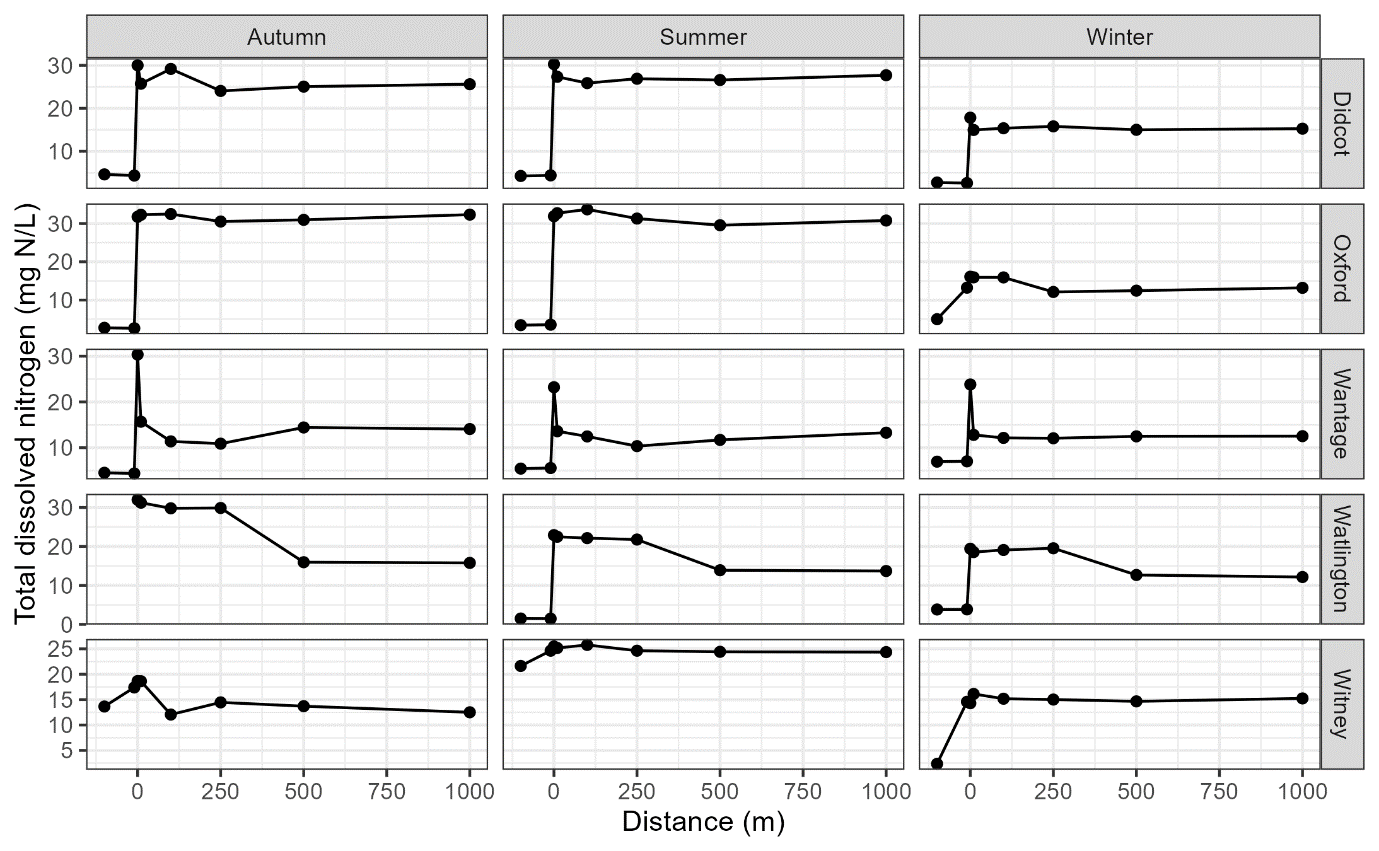


**Supplementary Figures 6D.** Dissolved nitrate (mg NO_3_/L).


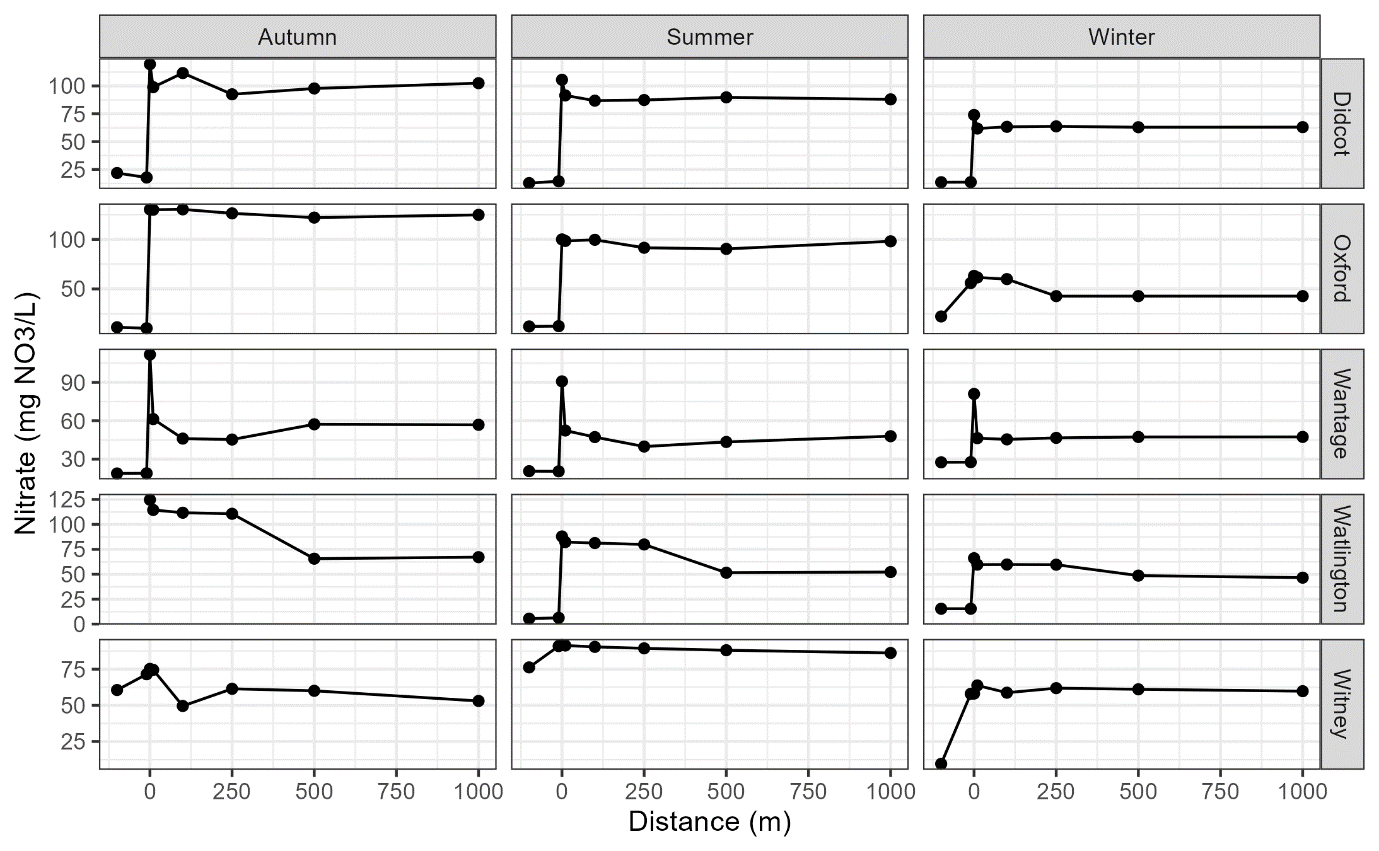


**Supplementary Figures 6E.** Dissolved nitrite (mg NO_2_/L).


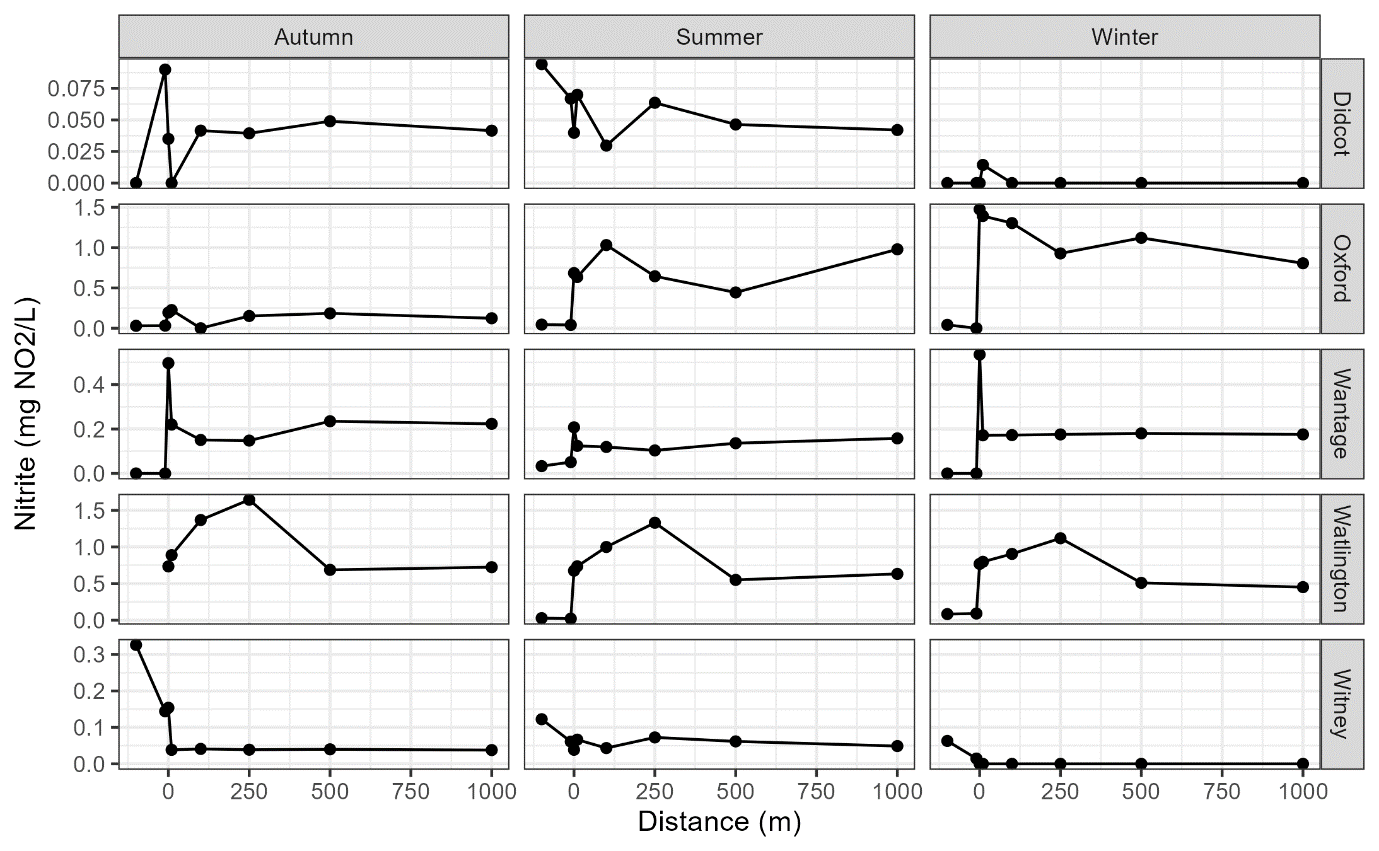


**Supplementary Figures 6F.** Dissolved chloride (mg/L).


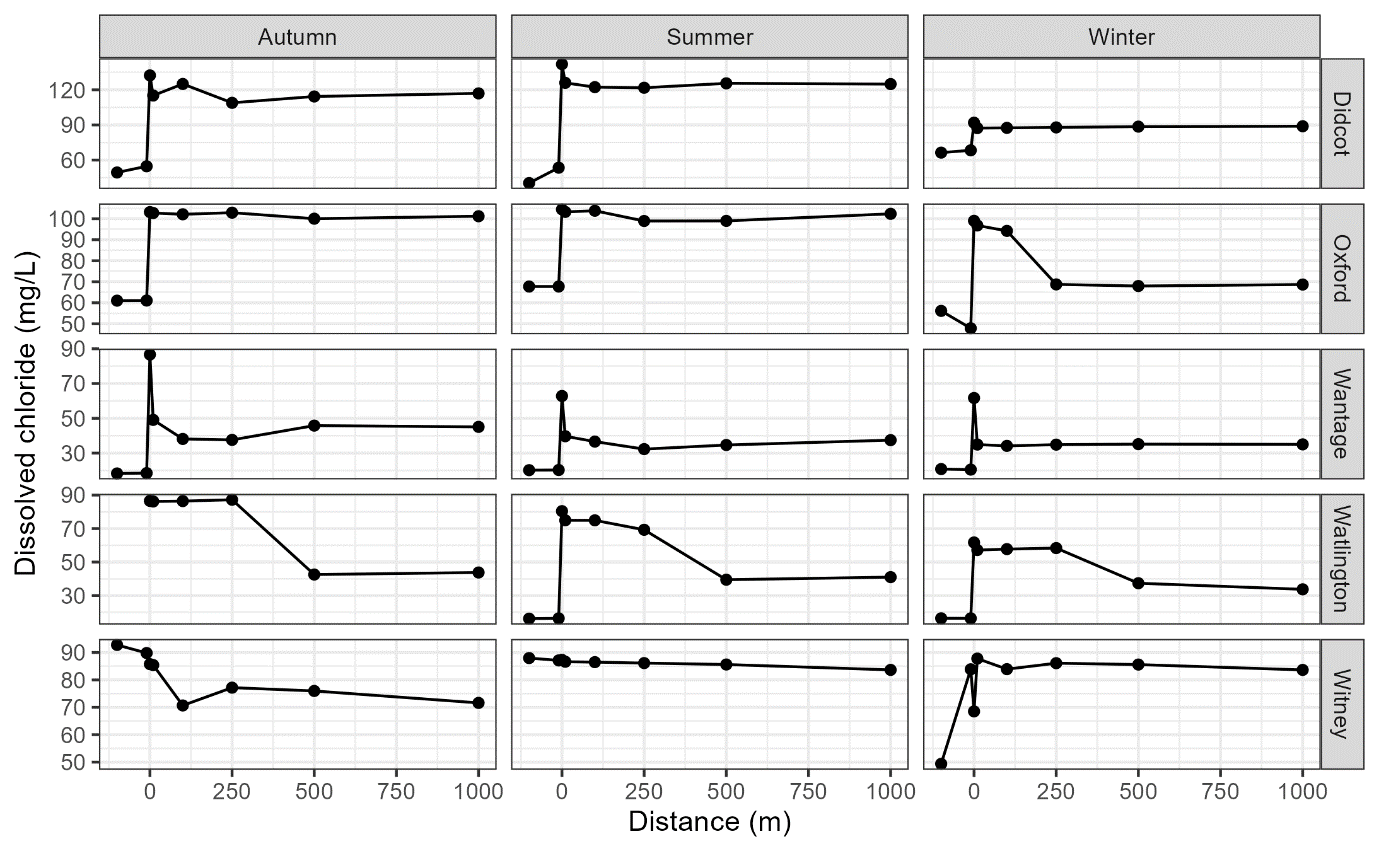


**Supplementary Figures 6G.** Dissolved fluoride (mg/L).


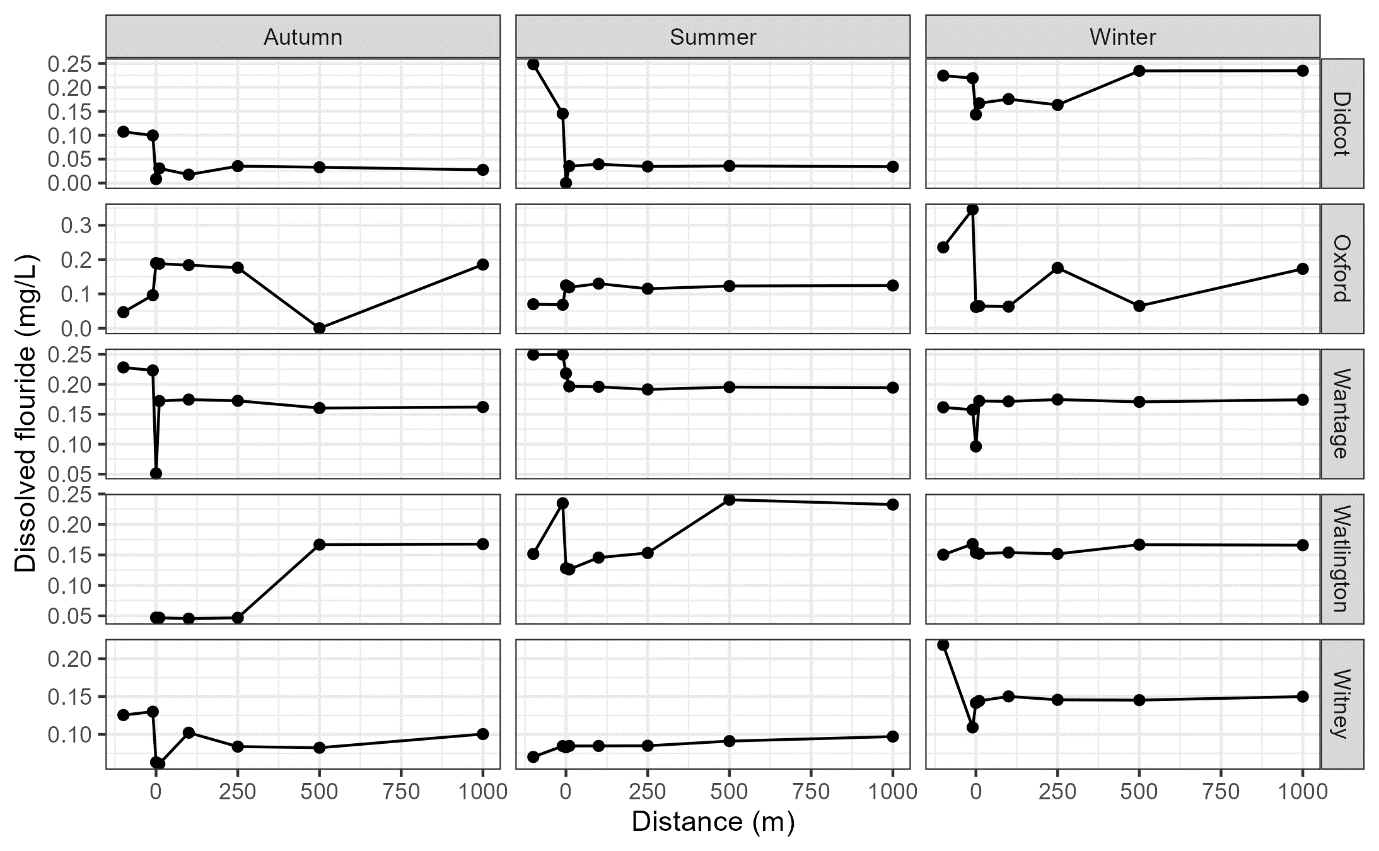


**Supplementary Figures 6H.** Dissolved ammonium (mg/L).


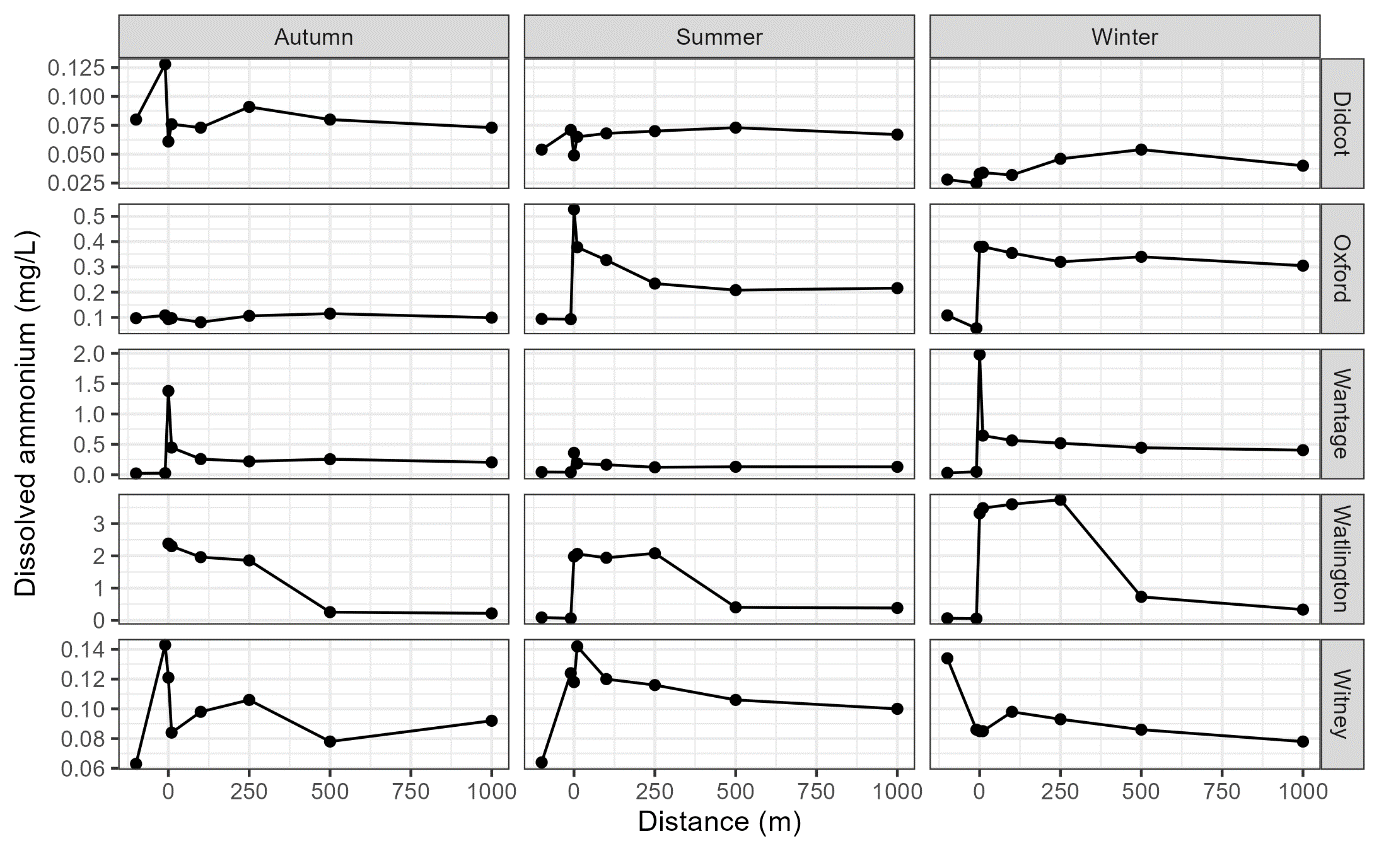


**Supplementary Figures 6I.** Soluble reactive phosphorus (µg/L).


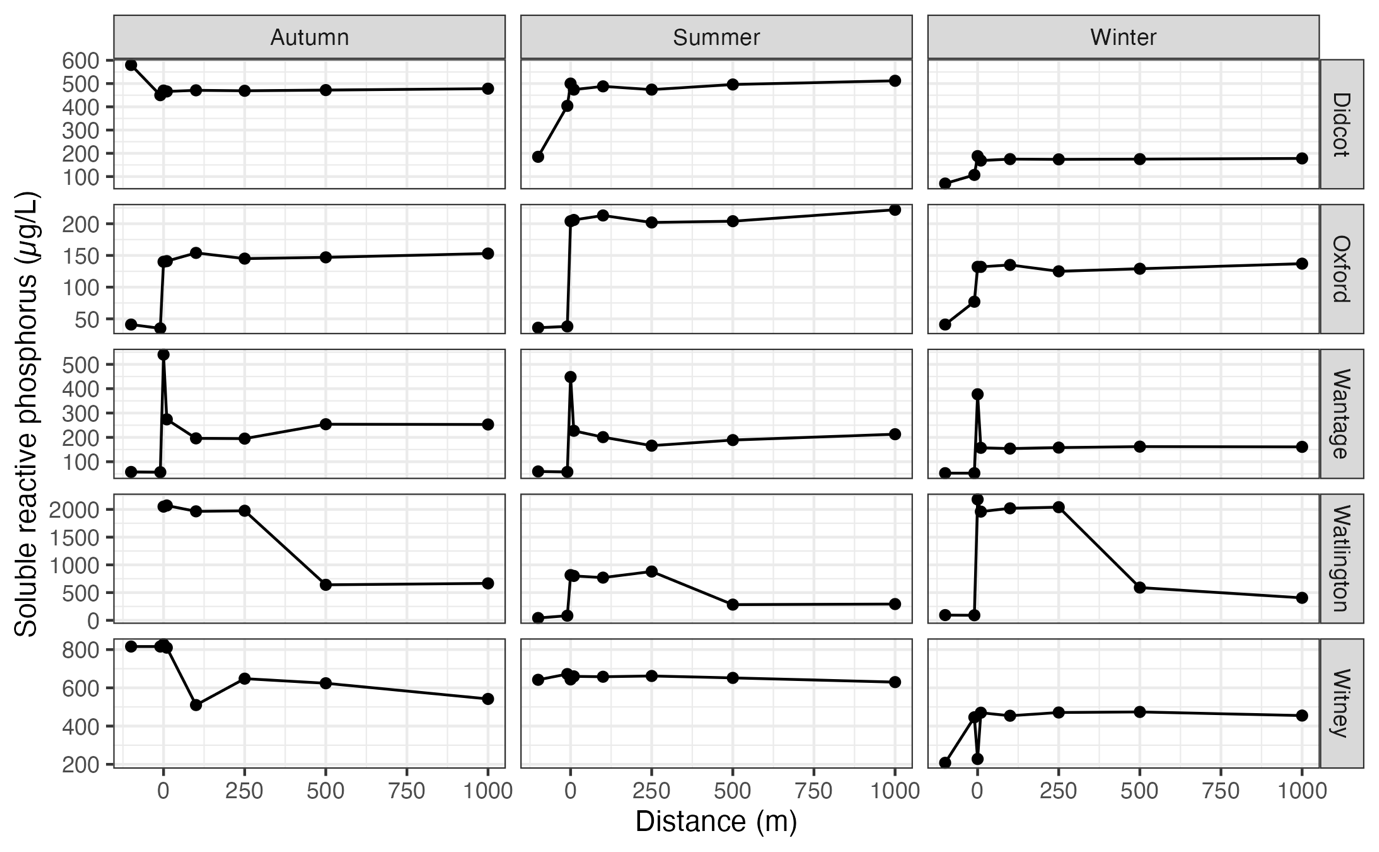


**Supplementary Figure S7 (next page)**. Aggregated water chemistry data across sampling sites and seasons. Stars on each plot indicate the results of a Wilcoxon signed-rank tests that compare the 100m upstream sites to the downstream sites. ns: p > 0.05, *: p <= 0.05, **: p <= 0.01, ***: p < 0.001, ****: p < 0.0001.


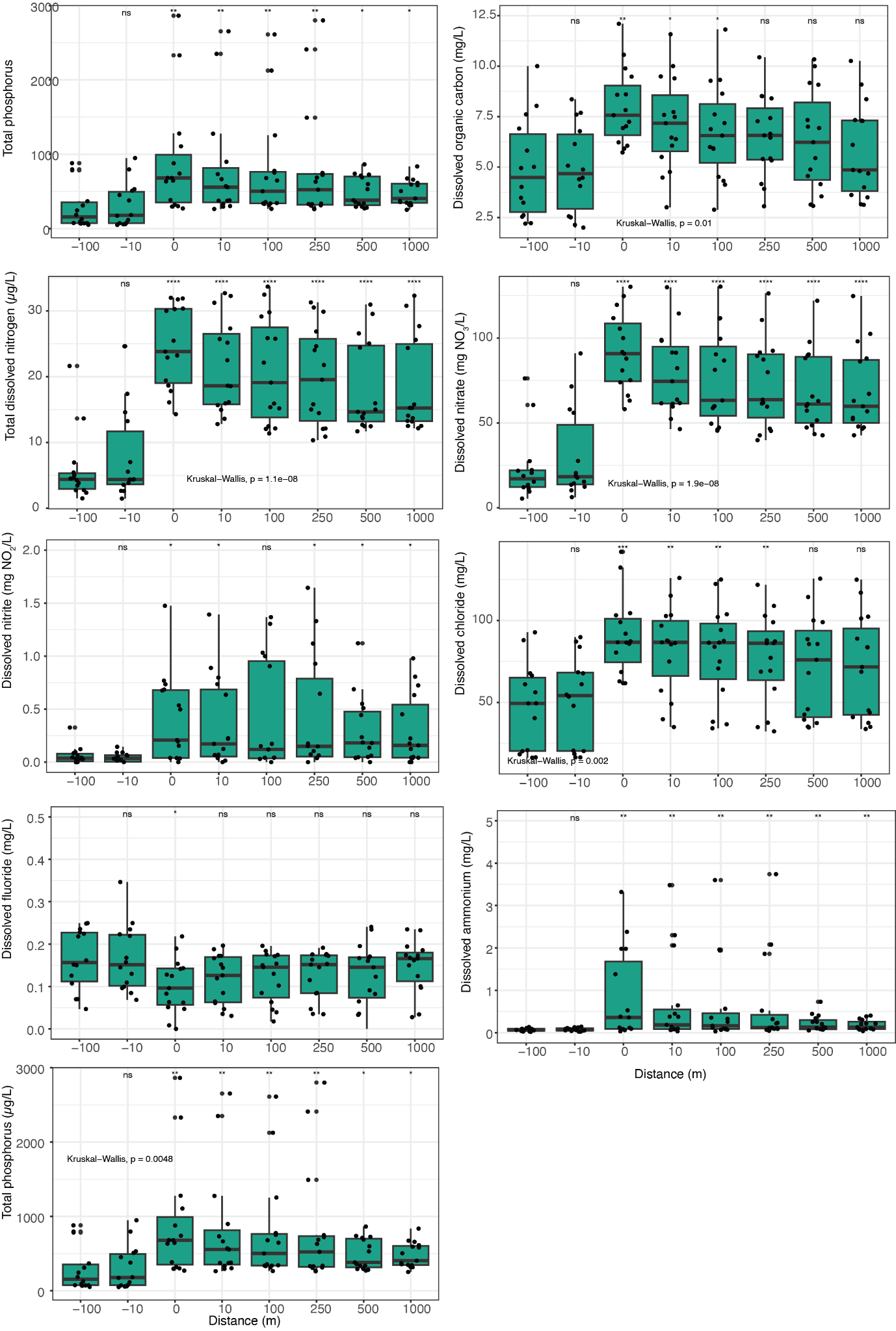


Supplementary Table 1. Percentage of all sample types with measured antibiotic concentrations above the AMR Industry Alliance PNECs for use in environmental risk assessment (AMR Industry Alliance, 2023). PNECs here represent the lowest of either PNEC-MICs (derived from Minimum Inhibitory Concentrations (MICs), protective of resistance promotion) (Bengtsson-Palme and Larsson 2016) or PNEC-ENVs (ecotoxicological, protective of the environment). Risk quotients (RQs) of concentrations of antibiotics measured in this study are also shown. RQs were calculated as follows: RQ=MEC/PNEC. RQs ≥1 indicate the concentration of this antibiotic poses a significant environmental risk and/or significant risk of AMR development (Sengar and Vijayanandan, 2022).

|  |  | Azithromycin | Clarithromycin | Metronidazole | Sulfadiazine | Sulfamethoxazole | Sulfanilamide | Trimethoprim |
| --- | --- | --- | --- | --- | --- | --- | --- | --- |
|  | PNEC (µg/L) | 0.03 | 0.25 | 0.13 | 11 | 6.6 | 230 | 0.5 |
| Upstream sites (n=5) | % above PNEC | 20% | 0% | 0% | 0% | 0% | 0% | 0% |
|  | Minimum RQ | 0 | 0 | 0 | 0 | 0 | 0 | 0 |
|  | Maximum RQ | 11.3 | 0.60 | 0 | 0 | 0 | 0 | 0 |
|  | Mean RQ | 2.27 | 0.12 | 0 | 0 | 0 | 0 | 0 |
| Downstream sites (n=5) | % above PNEC | 80% | 40% | 20% | 0% | 0% | 0% | 0% |
|  | Minimum RQ | 0 | 0.23 | 0 | 0 | 0 | 0 | 0.10 |
|  | Maximum RQ | 10.7 | 1.36 | 1.15 | 0 | 0.05 | 0 | 0.46 |
|  | Mean RQ | 4.90 | 0.74 | 0.38 | 0 | 0.02 | 0 | 0.30 |
| Effluent  (n=5) | % above PNEC | 80% | 20% | 0% | 0% | 0% | 0% | 0% |
|  | Minimum RQ | 0.29 | 0.36 | 0 | 0 | 0 | 0 | 0.10 |
|  | Maximum RQ | 3.00 | 1.88 | 0.92 | 0 | 0.05 | 0 | 0.38 |
|  | Mean RQ | 1.76 | 0.94 | 0.34 | 0 | 0.05 | 0 | 0.29 |

<https://www.amrindustryalliance.org/wp-content/uploads/2023/02/AMR-Table-1-Update-20230222_corrected.pdf>

Bengtsson-Palme, J., Larsson, D.G.J., 2016. Concentrations of antibiotics predicted to select for resistant bacteria: Proposed limits for environmental regulation. Environ Int 86, 140–149. <https://doi.org/10.1016/j.envint.2015.10.015>

Sengar, A., Vijayanandan, A., 2022. Human health and ecological risk assessment of 98 pharmaceuticals and personal care products (PPCPs) detected in Indian surface and wastewaters. Sci. Total Environ. 807, 150677. <https://doi.org/10.1016/j.scitotenv.2021.150677>
